# Supplementary material for: Anomalous thermo-osmotic conversion performance of ionic covalent-organic-framework membranes in response to charge variations
Source: Nat Commun. 2022 Jun 13;13:3386. doi: 10.1038/s41467-022-31183-w (PMC9192728; doi:10.1038/s41467-022-31183-w)
Supplement: Supplementary file 1 — Supplementary Information [file 41467_2022_31183_MOESM1_ESM.pdf]

Anomalous Thermo-osmotic Conversion Performance of Ionic Covalent-Organic-Framework Membranes in Response to Charge Variations

Weipeng Xian,[1] Xiuhui Zuo,[1] Changjia Zhu,[2] Qing Guo,[1] Qing-Wei Meng,[1] Xincheng Zhu,[1] Sai Wang,[1] Shengqian Ma,[2] Qi Sun[1,*]

[1]Zhejiang Provincial Key Laboratory of Advanced Chemical Engineering Manufacture Technology, College of Chemical and Biological Engineering, Zhejiang University, Hangzhou, 310027, China.
[2]Department of Chemistry, University of North Texas, 1508 W Mulberry St Denton, TX 76201, United States.

*Email: sunqichs@zju.edu.cn (QS)

Experimental Details

Transmembrane conductance measurement

The ionic current was recorded by a CHI660E (CH Instruments). A pair of Ag/AgCl electrodes was used to apply a transmembrane potential. The membrane was mounted between the two chambers (4 cm^3) of a homemade conductivity cell with a pore diameter of 1 mm. Both chambers were filled with an equal mole of KCl solution with the concentration ranging from 0.01 mM to 3 M. A scanning triangle voltage signal from -1 V to 1 V with a step voltage of 0.01 V and a period of 1 s was applied to record the I–V curves. The conductance values were derived from the slops of the resulting I–V curves.

Surface-charge-governed ion transport

The conductivities of the bulk values were measured by a conductivity meter. Considering that the bulk conduction is attributed to ions inside the bulk of the pore, surface conduction arises due to excess counterions close to the surface screening the surface charge, the so-called electric double layer. Accordingly, at high salt concentration the ion conduction is mainly determined by the bulk conductivity, but at low salt concentration, the surface conduction, determined by the surface conductivity, becomes dominant over the bulk conduction, leading to a saturation of the measured ion conductance. Therefore, we converted and normalized the measured transmembrane ion conductances according to the bulk conductivity collected at 3 M KCl.

Evaluation of transference number

For investigating the ion transport property of COF-EB_xBD_y/PAN, the ion current was recorded by CHI660E. To ignore the contribution of the redox potential of Ag/AgCl electrodes as a result of the inequable potential drop at the electrode-electrolyte interface, the conductivity cell with the pierced membrane separating three

chambers was used (4 cm^3), where the low concentration solution reservoirs were separated from a high concentration solution reservoir by the COF membrane and PAN, respectively (the effective testing area is 0.008 mm^2). The voltage was scanned with a step of 0.01 V s^{-1}. The ion transference number t_- of COF-EB_xBD_y/PAN was evaluated by determining the transmembrane diffusion potential (ϕ_{diff}). Herein, given that the redox potential of Ag/AgCl electrodes was eliminated during testing, ϕ_{diff} is equal to V_{oc}.

$$V_{oc} = \phi_{diff} = (2t_- - 1)\frac{RT}{F} ln\frac{a_{high}}{a_{low}} \tag{1}$$

The ion transference number t_- can be calculated with the following equation:

$$t_- = \frac{1}{2}\left(\frac{V_{oc}F}{RTln\frac{a_{high}}{a_{low}}} + 1\right) \tag{2}$$

where $a_{high}, a_{low}, V_{oc}, F, R,$ and T are the activities of ions in high concentration solution and low concentration solution, open circuit potential, Faraday constant, gas constant, and absolute temperature, respectively.

Evaluation of thermoelectric response of COF-EB_xBD_y/PAN

The thermoelectric response was recorded by a homemade setup (Figure 5b, inset, main text). A micro-ceramic heater (Zhuhai Huiyou Electronics, China) was employed to regulate the temperature of solutions. A direct current power (HSPY-60-5, Hanshengpuyuan, China) was connected to the heating rod to control the heating rate and temperature range. A pair of temperature microsensors (PT100, Tenghui Wenkong Instruments, China) was immersed in two solutions to measure the real-time temperature, which was recorded by a temperature sensor (THMA temperature recorder, Tenghui Wenkong Instruments, China). The measurement accuracy and working range of the temperature sensor are ± 0.1 K and −173.15−473.15 K, respectively. The transmembrane potential was synchronously collected by the CHI660E electrochemical workstation using two Ag/AgCl electrodes. The time resolutions of temperature recorder and transmembrane potential are both 1 s. For investigating the thermoelectric response property of the COF-EB_xBD_y/PAN membrane, a conductivity cell with the pierced membrane separating two chambers was used and both of the chambers were filled with 1 mM KCl solutions. A temperature gradient was induced by a brief heating of the chamber facing the COF active layer. The voltage variation relative to the initial state can be expressed as:

$$\Delta V_{oc}(T) = -2t_-\frac{R}{F}\Delta T ln\alpha \tag{3}$$

$$\Delta T = T_{low} - T_{high} \tag{4}$$

Osmotic energy harvesting

A pair of cation-selective membrane (Nafion®212) and anion-selective membrane (COF-EB_xBD_y/PAN) is mounted in a three-compartment conductive cell to harvest osmotic energy. The volume of each chamber is 4 cm^3 with a pore diameter of 0.1 mm. The high salinity solution (0.5 M NaCl) is placed in the middle chamber

and the low salinity solution (0.01 M NaCl) is set at the side chambers. Ag/AgCl electrodes were used to apply a transmembrane potential and measure the resulting current. The X-intercepts of the I–V plots represent the open-circuit voltage (V_{oc}) and the Y-intercepts of I–V plots present the short-circuit current (I_{sc}). The maximum output power can be calculated using

$$P_{max} = \frac{I_{sc}V_{oc}}{4} \tag{5}$$

$$PD_{max} = \frac{P_{max}}{S} \tag{6}$$

where P_{max}, PD_{max}, and S are the maximum power, maximum power density, and the effective working area of the COF membrane, respectively.

Evaluation of the thermo-osmotic power production

The thermo-osmotic power production of COF-EB_xBD_y/PAN was evaluated using a homemade setup. A micro-ceramic heater (Zhuhai Huiyou Electronics, China) was employed to regulate the temperature of solutions. A direct current power (HSPY-60-5, Hanshengpuyuan, China) was connected to the heating rod to control the heating rate and temperature range. A pair of temperature microsensors (PT100, Tenghui Wenkong Instruments, China) was immersed in two solutions to measure the real-time temperature, which was recorded by a temperature sensor (THMA temperature recorder, Tenghui Wenkong Instruments, China). The transmembrane potential was synchronously collected by the CHI660E electrochemical workstation using a pair of Ag/AgCl electrodes. A conductivity cell with the pierced membrane separating three fluidic chambers was used, where the chambers filled with 0.01 M NaCl solution were separated from the chamber filled with 0.5 M NaCl solution by the COF membrane and Nafion®212, respectively, with the COF active layer facing the low concentration side. The volume of each chamber is 4 cm^3 with a pore diameter of 0.1 mm. A temperature gradient was induced by a brief heating of the 0.01 M NaCl solution. The voltage variation relative to the initial state is dependent on both temperature and activity gradient, which can be calculated using the following equation:

$$V_{oc} = -2t_{-}\frac{R}{F}(T_{low}\ln a_{low} - T_{high}\ln a_{high}) \tag{7}$$

where T_{high}, and T_{low} are the temperatures of high concentration and low concentration solutions. When the temperature of low concentration solution was changed (the temperature of solution in high concentration solution remained unchanged), the voltage variation relative to the initial state can be expressed as:

$$\Delta V_{oc}(T) = -2t_{-}\frac{R}{F}\Delta T ln a_{low} \tag{8}$$

The maximum output power density can be calculated using equations 5 and 6.

Stability evaluation

A pair of a cation-selective membrane (Nafion®212) and anion-selective membrane (COF-EB_1BD_2/PAN) is mounted in a three-compartment conductive cell to harvest osmotic energy. The high salinity solution (0.5 M NaCl) is placed on the middle reservoir, and the low salinity solution (0.01 M NaCl) is set at the side reservoirs, with the COF active layer facing the low concentration side using the setup for the evaluation of thermo-osmotic power production. The electrolyte solutions were replenished with fresh solutions at each cycle. The temperature gradient of 40 K was induced by a brief heating of the 0.01 M NaCl solution. I–V plots of the RED device were recorded every 24 h. The initial V_{oc}, I_{sc}, and power density are 167.7 mV, 35.7 μA, and 191 W m^{-2}, which are maintained at 163.7 mV, 34.8 μA, and 181 W m^{-2} after 14 d, respectively.

Numerical simulations

The numerical simulation was performed based on coupled Poisson and Nernst-Planck equations by setting appropriate boundary parameters using a commercial finite-element software package COMSOL (version 5.2). The Nernst-Planck equation (9) defines the flux of each ion species in the presence of concentration gradient, which describes the transport character of charged nanochannels. The ion concentration induced electrical potential can be described by Poisson equation (10). When the system reaches a stationary regime, the ion flux should conform to the steady-state continuity equation (11).

$$j_i = D_i\left(\nabla c_i + \frac{z_i F\, c_i}{RT}\nabla\phi\right) \tag{9}$$

$$\nabla^2\phi = -\frac{F}{\varepsilon}\sum z_i c_i \tag{10}$$

$$\nabla \cdot j_i = 0 \tag{11}$$

Where j_i, c_i, z_i, and D_i are the ionic flux, ion concentration, charge number of each ionic species, and diffusion coefficient. ϕ and ε represent the electrical potential and the dielectric constant of medium. T, F, and R are the absolute temperature, Faraday constant, and universal gas constant, respectively. The diffusion coefficients for cations and anions are 1.334×10^{-5} and 2.032×10^{-5} cm^2 s^{-1} (NaCl was used as the electrolyte).

For simplification, a 1.5-nm-width and 200-nm-length rectangle nanochannel was used to simulate the channels inside the COF-EB_xBD_y/PAN. To decrease the effect of entrance/exit mass transfer resistances on the overall ionic transport, two electrolyte reservoirs are introduced. The external potential is applied on the boundary w_1, and w_2 offered the reference potential. The boundary conditions for the electrical potential and ion flux are shown as below:

$$\vec{n} \cdot \nabla\phi = -\frac{\sigma}{\varepsilon} \tag{12}$$

$$\vec{n} \cdot j = 0 \tag{13}$$

The physical quantity σ represents the surface charge density of the channel walls. The ionic current can be calculated by

$$I = \iint j_i \, ds = -\iint D_i \left(\nabla c_i + z_i c_i \frac{F}{RT} \nabla \phi \right) ds \tag{14}$$

In addition to the concentration gradient, temperature gradient, as an external driving force, also drives the motion of solutes. Taking the thermal potential inside a nanochannel into account, the numerical model has to consider both mass and heat transfers. To facilitate the calculation, the diffusion coefficients of Na^+ and Cl^- were fixed during heating. Therefore, we introduce the Nernst-Plank equation into the model to describe ion transport and concentration profiles under a temperature gradient,

$$j_i = D_i \left(\nabla c_i + z_i c_i \frac{F}{RT} \nabla \phi - c_i u \right) \tag{15}$$

$$I = \iint j_i \, ds = -\iint D_i \left(\nabla c_i + z_i c_i \frac{F}{RT} \nabla \phi - c_i u \right) ds \tag{16}$$

where u is the velocity induced by thermo-osmosis and thermoelectric effect, which can be formulated as follow[1],

$$u = -\frac{\varepsilon \zeta^2}{3\eta} - \frac{\varepsilon \zeta \psi_0}{\eta} \frac{\nabla T}{T} \tag{17}$$

where ζ is the zeta potential; η is the viscosity of solvent; ψ_0 is the bulk electric potential.

For the power generation calculations, a concentration gradient (c_{high}/c_{low} = 0.5 M/0.01 M) is employed and no external potential is applied. The corresponding diffusion current can be attained by:

$$I_{diff} = \left| |I_+| - |I_-| \right| \tag{18}$$

$$\phi_{diff} = \phi_{high} - \phi_{low} \tag{19}$$

Ion Transmission Activation Energy Measurement

The membrane was mounted between the two chambers of a symmetric H-type electrochemical cell with a pore diameter of 0.1 mm and both chambers were filled with different concentrations of NaCl aqueous solutions (4 cm^3 for each). The COF layer was in contact with the low concentration side. Ag/AgCl electrodes were used to apply a transmembrane potential. A scanning triangle voltage signal from 0.2 V to -0.02 V with a step voltage of 0.01 V and a period of 1 s was applied to record the I–V curves. The ionic currents of the systemes at various temperatures were recorded by a CHI660E. The conductance values were derived from the slops of the resulting I–V curves. The ion transmission activation energies were calculated by the Arrhenius equation:

$$G = Ae^{-\frac{E_a}{RT}} \quad (20)$$

whereby G, A, E_a, R, and T represnet the conductance, preexponential factor, activation energy, gas constant, and temperature, respectively.

Supplementary Table 1 │ Zeta potentials for the COF-EB_xBD_y/PAN membranes and the free-standing COF-EB_xBD_y membranes collected at pH=6.5.

Composite membrane	Zeta potential (mV)[a]
COF-BD/PAN	10.9
COF-EB_1BD_5/PAN	13.0
COF-EB_1BD_3/PAN	18.2
COF-EB_1BD_2/PAN	20.7
COF-EB/PAN	25.8
Free-standing membrane	
COF-BD	5.4
COF-EB_1BD_5	8.9
COF-EB_1BD_3	9.2
COF-EB_1BD_2	10.3
COF-EB	21.9

[a]The zeta potentials for these membranes were calculated using Z(COF-EB_xBD_y/PAN) – Z(PAN), whereby Z(COF-EB_xBD_y/PAN) is the zeta potential of the composite COF-EB_xBD_y/PAN membranes, and Z(PAN) is the zeta potential of PAN (-51.1 mV), respectively.

Supplementary Table 2 │ The content of bromine species in the free-standing COF-EB_xBD_y/PAN membranes.

Membrane	Theoretical values (mmol g^{-1})	Elemental analysis (mmol g^{-1})
COF-BD	--	--
COF-EB_1BD_5	0.026	0.017
COF-EB_1BD_3	0.049	0.031
COF-EB_1BD_2	0.066	0.042
COF-EB	0.250	0.250

Supplementary Table 3 | The charge density in the COF-EB_xBD_y/PAN membranes calculated according to the results of elemental analysis.

Membrane	Charge density (C m^{-2})
COF-BD	0
COF-EB_1BD_5	0.02
COF-EB_1BD_3	0.03
COF-EB_1BD_2	0.04
COF-EB	0.12

Because of the negligible charge screening ability of COF-BD, we assume that the charge density of COF-BD is 0 C m^{-2} to facilitate the calculation. The charge density of COF-EB_xBD_y was calculated using the following equations:

$$n = \frac{n_e}{n_t} \times 100$$

$$\xi = \frac{x}{x+y} \times 100\%$$

where n_e (%) and n_t (%) is the elemental analysis value and theoretical value of Br^- species in the membrane respectively. n (%) is the ratio of actual charge density to theoretical charge density, and ξ (%) is the ratio of quaternary ammonium cation:

$$\sigma = n\xi \frac{Ne}{S}$$

where N, e (C), S (m^2), and σ (C m^{-2}) are the number of quaternary ammonium cation in COF-EB, the charge quantity, effective area of nanochannels, and surface charge density of COF-EB_xBD_y, respectively.

Supplementary Table 4 | Atomistic coordinates for the eclipsed mode of the COF-BD membrane optimized using the Forcite method (space group P-1, a=27.2612 Å, b=27.3574 Å, c= 4.7452 Å, α=β=90° and γ= 120.5111°).

Atom	*x/a*	*y/b*	*z/c*
C1	0.55159	0.77308	0.16127
C2	0.51856	0.71377	0.14610
N3	0.46394	0.68535	-0.02715
O4	0.20989	0.60366	-0.45581
H5	0.44201	0.64104	-0.01744
C6	0.04960	0.02090	1.53287
C7	0.01812	0.05103	1.47656
C8	0.35047	0.62416	-0.36890
C9	0.38306	0.68606	-0.31119
C10	0.43560	0.71375	-0.17190
O11	0.93019	0.04157	1.63765
H12	0.45396	0.75898	-0.13611
C13	0.21046	0.76552	0.29639
C14	0.26553	0.79710	0.15557
N15	0.29340	0.77068	-0.01983
O16	0.37360	0.59812	-0.47218
H17	0.33766	0.79427	-0.02975
C18	0.95623	0.01843	1.53435
C19	0.92611	0.95677	1.47340
C20	0.35293	0.71753	-0.36755
C21	0.29120	0.68843	-0.30323
C22	0.26419	0.71386	-0.16237
O23	0.93573	0.87787	1.62135
H24	0.21856	0.68847	-0.13565
C25	0.21062	0.42915	0.21595
C26	0.18228	0.45982	0.16843
N27	0.20801	0.51379	-0.01294
O28	0.37883	0.76640	-0.47642
H29	0.18548	0.53557	-0.00395
C30	0.95881	0.92735	1.52441
C31	0.02050	0.95917	1.46860
C32	0.25937	0.62674	-0.35893
C33	0.28880	0.59405	-0.30791

C34	0.26394	0.54136	-0.17080
O35	0.09848	0.04680	1.64165
H36	0.29010	0.52199	-0.15105
C37	0.12705	0.43700	0.30409
C38	0.10000	0.38403	0.47482
C39	0.12777	0.35243	0.51440
C40	0.18378	0.37640	0.38447
C41	0.09907	0.29510	0.68888
C42	0.03951	0.26159	0.70179
C43	0.13120	0.27270	0.84097
C44	0.10513	0.21955	1.00429
N45	0.01740	0.13190	1.19260
C46	0.04581	0.10356	1.33726
H47	0.10500	0.46062	0.27785
H48	0.20710	0.35406	0.39988
H49	-0.03238	0.18277	0.86835
H50	0.17688	0.29662	0.84822
H51	0.13240	0.20613	1.12565
H52	-0.02691	0.10997	1.18291
H53	0.09104	0.12192	1.30138
C54	0.01340	0.20802	0.86486
C55	0.04583	0.18652	1.01937
C56	0.54007	0.68136	0.30058
C57	0.59364	0.70746	0.46368
C58	0.62714	0.76702	0.47666
C59	0.60473	0.79915	0.32461
C60	0.68447	0.79573	0.65115
C61	0.71608	0.76796	0.69069
C62	0.70844	0.85173	0.78111
C63	0.76120	0.87857	0.94964
N64	0.84585	0.87598	1.17847
C65	0.87341	0.93191	1.33631
H66	0.51483	0.63558	0.29703
H67	0.79269	0.77297	0.88764
H68	0.68609	0.87504	0.76574
H69	0.77652	0.92112	1.04443
H70	0.86763	0.85346	1.16949
H71	0.85405	0.95806	1.31655

C72	0.76906	0.79502	0.86143
C73	0.79187	0.85024	0.99711
C74	0.29461	0.85601	0.19472
C75	0.26857	0.88337	0.35835
C76	0.21285	0.85208	0.49588
C77	0.18477	0.79253	0.46718
C78	0.18413	0.88080	0.66941
C79	0.21543	0.93652	0.80691
C80	0.12458	0.85273	0.69813
C81	0.09758	0.87842	0.86892
N82	0.10274	0.96136	1.18510
C83	0.04593	0.93216	1.32771
H84	0.33717	0.88086	0.08830
H85	0.14360	0.76596	0.58737
H86	0.21293	1.00512	1.07695
H87	0.09800	0.81156	0.57796
H88	0.05190	0.85555	0.87593
H89	0.12633	1.00562	1.19499
H90	0.02054	0.88654	1.30103
C91	0.18807	0.96256	0.97055
C92	0.12916	0.93349	1.00972
H93	0.18759	0.71985	0.28940
H94	0.25317	0.44449	0.12120
H95	0.60756	0.68067	0.58939
H96	0.70101	0.72598	0.58200
H97	0.29210	0.92907	0.36658
H98	0.26114	0.96004	0.79867
H99	0.05801	0.36895	0.58349
H100	0.01271	0.27550	0.57605
H101	0.53816	0.80034	0.03998
H102	0.62865	0.84483	0.31741

Supplementary Table 5 │ Osmotic/thermo-osmotic energy harvesting performance of representative RED systems in the literature.

Materials	Charge density ($C\ m^{-2}$)	System	Power density ($W\ m^{-2}$)	Testing area (mm^2)	Resistance (kΩ)	ΔT (K)
**2D materials**						
$Ti_3C_2T_x$ MXene membranes[2]		0.5 M/0.01 M NaCl	4.6	0.2	5	
MXene/Kevlar nanofiber composite[3]	0.02	0.5 M/0.01 M NaCl	4.1	0.03	30	
$Ti_3C_2T_x$ MXene[4]		1 M/0.001 M KC	20.85	0.0163	14	
$Ti_3C_2T_x$ MXene/BN membrane[5]		0.5 M/0.01 M	0.8(6.2[a])	7.07 cm^2	60	60
Graphene oxide membrane[6]	0.15	0.5 M/0.01 M NaCl	0.77	0.2	10	
Vertically transported graphene oxide[7]		0.5 M/0.01 M NaCl	10.6	0.00355	100	
Graphene reverse electrodialysis device[8]	0.02	1 M/0.001 M NaCl	8	1.5 μm^2	130000	
GO/Silk nanofiber membrane[9]		0.5 M/0.01 M NaCl	5.07 (9[a])	0.03	35	40
2D/3D-GO/polymer[10]		0.5 M/0.01 M NaCl	0.76		300	
Polymeric-C_3N_4[11]		0.1 M/0.1 mM KCl	0.21	1.5	8.9	
Nanocomposite membranes (BN)[12]		0.5 M/0.01 M NaCl	5.9	0.03		
BN nanopore membrane[13]	0.0323	1000 mM/1 mM KCl	0.17	0.1		
Single transmembrane BN nanotube[14]	0.1	1 M/0.1 mM KCl	4000	10000 nm^2	54348	
**Inorganic materials**						
Silk-based AAO hybrid membranes[15]		0.5 M/0.01 M NaCl	2.86	0.03	23	
AAO ionic diode membrane[16]	0.06	0.5 M/0.01 M NaCl	3.46	0.03	10	
Heterogeneous BCP/AAO[17]		0.5 M/0.01 M NaCl	2.94	0.03	46	
CMWs/AAO membrane[18]		0.5 M/0.01 M NaCl	2.78	0.03	10	
Silica/AAO heterostructure membranes[19]		0.5 M/0.01 M NaCl	4.5	0.03	10	
AAO nanochannels[20]		0.1 mM/0.1 mM KCl[a]	25.5		50	30
Solar thermoelectric nanofluidic[21]	-0.0052		4.5×10-5[a]			35
Ultrasmall silica nanochannels[22]		0.5 M/0.01 M NaCl	1.0 (1.404[a])	0.0327	20	10
Silica nanochannels[23]	0.002	1 M/0.1 mM KCl	7.7			
Ultrathin silica isoporous membrane[24]	0.015	0.5 M/0.01 M NaCl	0.024	0.25	220	
Nanofluidic crystal silicon[25]		0.1 M/0.1 mM KCl	2.82	0.00532	1300	
Silica[26]		300 mM/0.1 mM KCl	3.9	55.7 μm^2	14000	
Black phosphorus membranes[27]	0.002	0.6 M/0.004 M NaCl	4.7	0.03	40	
2D metallic MoS_2[28]		0.5 M/0.01 M NaCl	6.7	0.03	23	

Single-layer MoS_2[29]	0.0469	1 M/1 mM KCl	1000000	100 nm^2	9400	
Montmoril-modification lamellae[30]		1 M/1 mM KCl	0.15 (0.34[a])	0.785	10	30
Nanokaolinite membranes[31]		0.1 M/0.001 M KCl	0.18	0.2	250	
**Polymers**						
Janus 3D porous membrane[32]	0.05	0.5 M/0.01 M NaCl	2. 66		10	
PVA gel electrolytes[33]	0.005	100 mM/0.1 mM KCl	0.624	0.03	100	
Three-dimensional hydrogel interface[34]		0.5 M/0.01 M NaCl	5.06	0.03	40	
TOCNs/PET[35]	-0.012	0.5 M/0.01 M NaCl	0.96	9	100	
Janus membranes (PSS)[36]		0.5 M/0.01 M NaCl	2.04	0.03	20	
Single-ion-selectivenanopores (PET)[37]		1 M/1 mM KCl	20-2600	225 nm^2	16000	
Paper channel [38]		0.3 M/0.0001 M KCl	0.00275	20	10	
Heterogeneous membrane (PET)[39]		0.5 M/0.01 M NaCl	0.35		150	
Hydrogel-based nanofluidics[40]		0.5 M/0.01 M NaCl	4.08	0.03	25	
Cyto-compatible asymmetric polypyrrole[41]		0.5 M/0.01 M NaCl	0.087		5	
Poly(ether sulfone) bipolar membrane[42]		0.5 M/0.01 M NaCl	6.2	0.03	10	
PES/SPES nanochannel[43]		0.5 M/0.001 M NaCl	2.48		30	
Polyelectrolyte-filled mesopores[44]		500 mM/1 mM KCl	945	0.123 μm^2	54000	
SPEEK[45]	0.0423	0.5 M/0.01 M NaCl	5.8	0.03	20	
Nanochannel array membrane (PEI)[46]		0.5 M/0.01 M NaCl	13.2	0.008	10	
PCTE membrane[47]	-0.0065	10 mM/1 mM KCl	74.8 nW (177.5[a])			25
PET membrane[48]	-0.06	1 M/1 mM KCl	56.22% enhanced[a]			30
SPEEK/PES blend membrane[49]		0.5 M/0.01 M LiBr	9.26 (16.50[a])	0.2	30(7[a])	30
Alternating cells[50]		35.4 g L^{-1}/0.56 g L^{-1} NaCl	0.46	4 cm^2	0.005	
Ion exchange membrane pairs[51]		0.5 M/0.01 M NaCl	0.26		0.082	
Ion exchange membrane[52]		30 g/1 g NaCl	1.2			
Ion exchange membranes[53]		0.6 M/1.5 mM	3.5			
RED-MED system[54]		4.5 M/0.05 M NaCl	4 (14[a])	8.8×10^4	132	80
**Framework materials**						
Polymer/MOF[55]	0.01	0.5 M/0.01 M NaCl	2.87	0.03	6.5	
COF-TpPa-SO_3H[56]		0.5 M/0.01 M NaCl	5.9	0.03	23	
MOFs membrane[57]		0.5 M/0.01 M NaCl	2.96	0.03	50	
PyPa-SO_3H/SANF membranes[58]		0.5 M/0.01 M NaCl	8.3	20.74	5	
**Biomaterials**						
Silk fibroin membrane[59]	0.008	5 M/0.01 M NaCl	21.66	0.03	4	
Bacterial cellulose membranes[60]	0.0031	0.5 M/0.01 M NaCl	0.23	1.8	7	

Ionized wood membrane[61]	0.0031	0.6 M/0.01 M NaCl	0.00514	100		
**This work**						
COF-BD/PAN	0	0.5 M/0.01 M NaCl	32 (57[a])	0.008	12	40
COF-EB_1BD_5/PAN	0.02	0.5 M/0.01 M NaCl	77 (134[a])	0.008	8	40
COF-EB_1BD_3/PAN	0.03	0.5 M/0.01 M NaCl	99 (183[a])	0.008	7	40
COF-EB_1BD_2/PAN	0.04	0.5 M/0.01 M NaCl	113 (215[a])	0.008	7	40
COF-EB/PAN	0.12	0.5 M/0.01 M NaCl	110 (218[a])	0.008	7	40
COF-EB_1BD_2/PAN	0.04	0.5 M/0.01 M NaCl	33 (63[a])	0.03	7	40
COF-EB/PAN	0.12	0.5 M/0.01 M NaCl	31 (65[a])	0.03	7	40

[a] The values in parentheses refer to the output power density in the presence of a temperature difference.

Supplementary Table 6 │ Seebeck coefficient of representative materials in the literature.

Materials	Seebeck coefficient (mV K^{-1})
Thermoelectric fibers[62]	0.15
Graphene[63]	0.02
Woven thermoelectric fibers[64]	0.064
PEDOT: PSS film[65]	0.043
Bismuth telluride micrograins[66]	0.24
SnSe crystals[67]	0.22
Hole-doped tin sulfide crystals[68]	0.21
$Cd_{0.99}Ag_{0.01}Sb$[69]	0.26
PET conical nanochannels[70]	0.44
SIM/PET[70]	0.71
PAN[71]	0.51
Asy-AAO-Au[72]	0.76± 0.17
Shark[73]	0.40
TpTag-COF/PAN[74]	1.16
**This work**	**1.09**

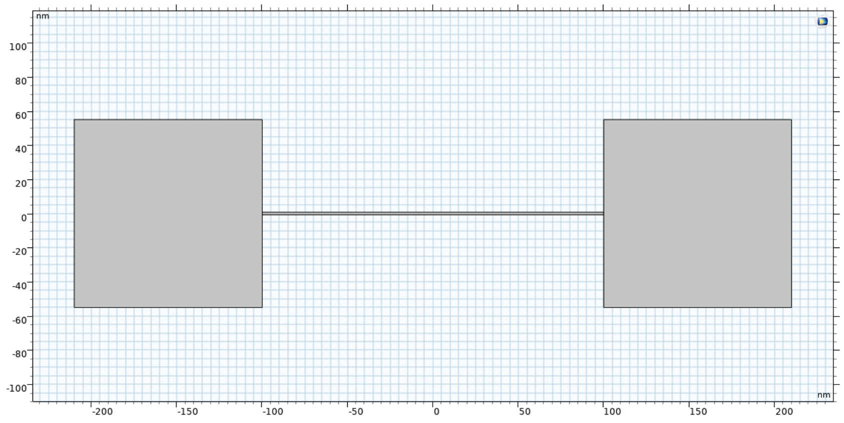

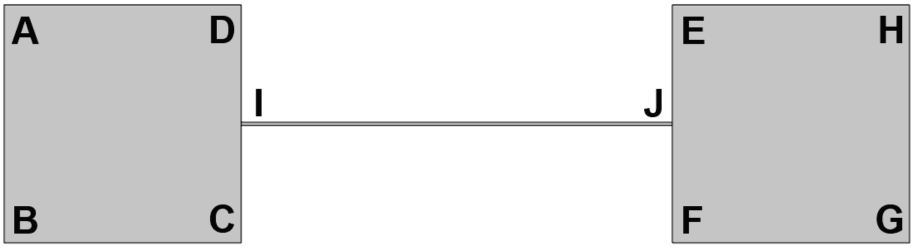

Supplementary Table 7 │Boundary conditions for planar model.

Surface	Electric Potential	Ion Transport	Flow Field	Heat Transfer
AB	Ground	Low concentration	Pressure=0	T=298.15+δT
BC, DA, FG, EH	Zero charge $\vec{n}\cdot\nabla\phi = 0$	No flux $-\vec{n}\cdot\boldsymbol{J}_i = 0$	Slip boundary	Thermal insulation
GH	Constant voltage $\phi = \mathrm{V}$	High concentration	Pressure =0	T=298.15
I-J	Surface charge density $-\vec{n}\varepsilon_0\varepsilon_r\nabla\phi = \rho_s$	No flux $-\vec{n}\cdot\boldsymbol{J}_i = 0$	No slip	Thermal insulation
DC, EF	Zero charge $\vec{n}\cdot\nabla\phi = 0$	No flux $-\vec{n}\cdot\boldsymbol{J}_i = 0$	No slip	Thermal insulation

Mesh independence check. The model is meshed using quadratic elements with a finer mesh size in the region of the electrical double layer[75]. Prior to the detailed simulation process, a grid independence test was performed. Based on the simulation results, the mesh number was set in the planar model as 1450、6888、10584、17450 and 42285 for 1, 5, 9, 17 and 40 channels respectively.

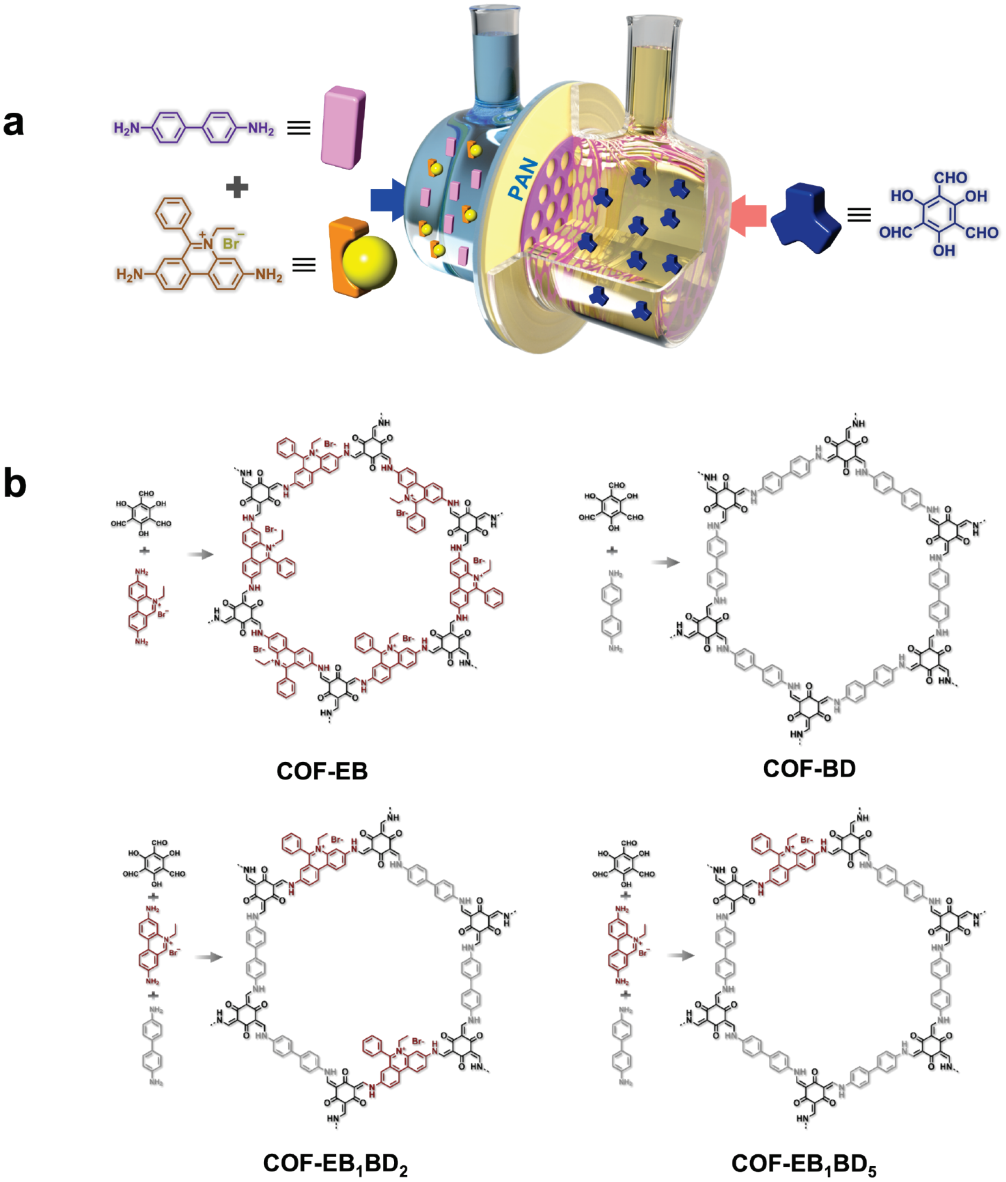

Supplementary Figure 1 | Fabrication of membranes. (a) Schematic illustration of the set-up used for the fabrication of COF-EB_xBD_y/PAN *via* interfacial polymerization. BD and EB were dissolved in TsOH aqueous solution, and Tp was dissolved in dichloromethane. (b) Chemical structures of the COF membranes.

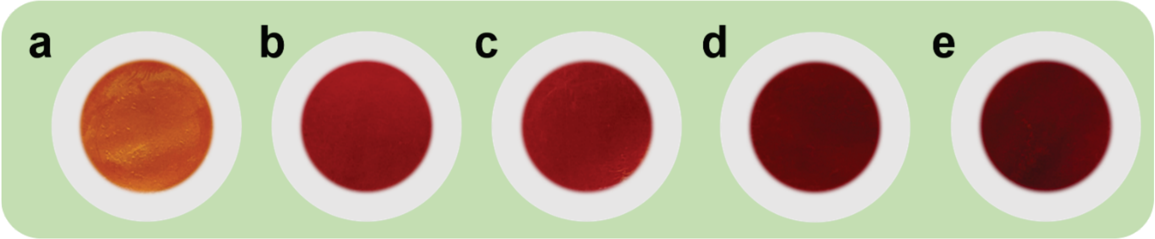

Supplementary Figure 2 | Digital photos. (a) COF-BD/PAN, (b) COF-EB_1BD_5/PAN, (c) COF-EB_1BD_3/PAN, (d) COF-EB_1BD_2/PAN, and (e) COF-EB/PAN.

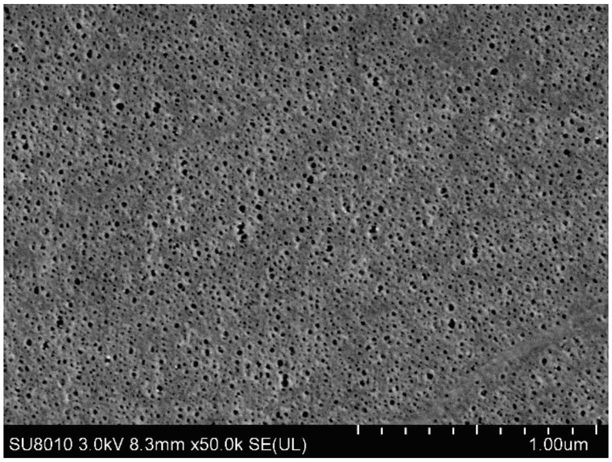

Supplementary Figure 3 │ Top-view SEM image of the PAN membrane.

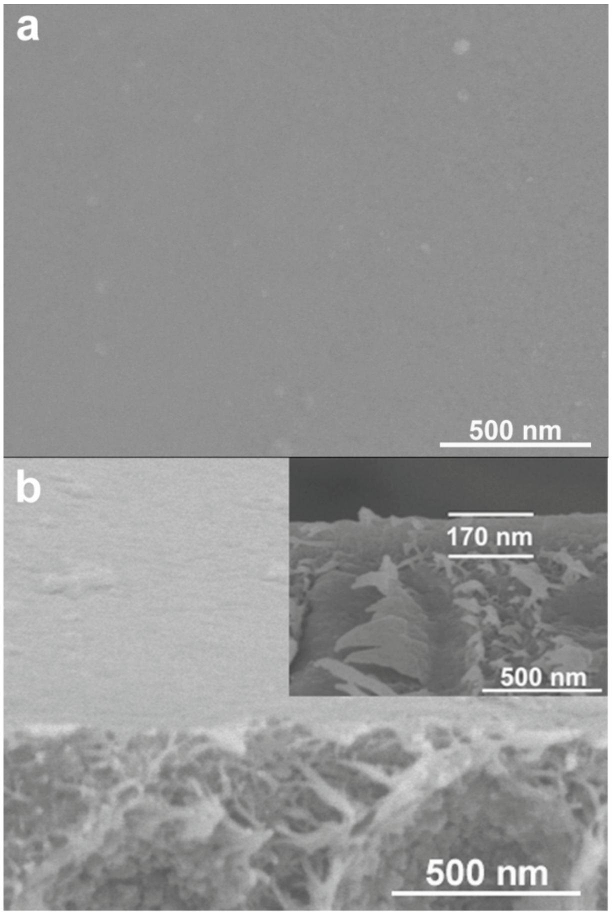

Supplementary Figure 4 | SEM images. (a) top view and (b) cross section-view SEM images of COF-BD/PAN (inset: the thickness of the membrane).

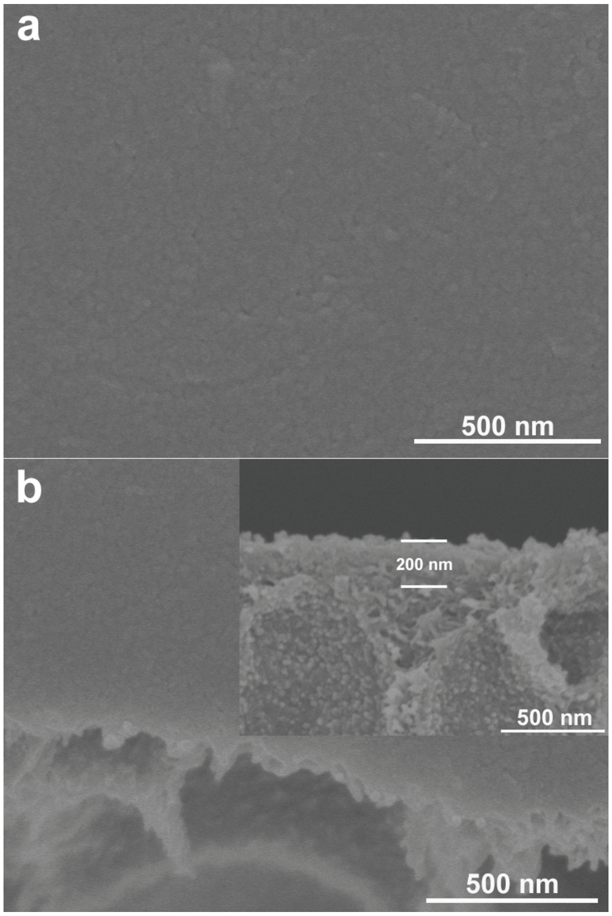

Supplementary Figure 5 | SEM images. (a) top view and (b) cross section-view SEM images of COF-EB_1BD_5/PAN (inset: the thickness of the membrane).

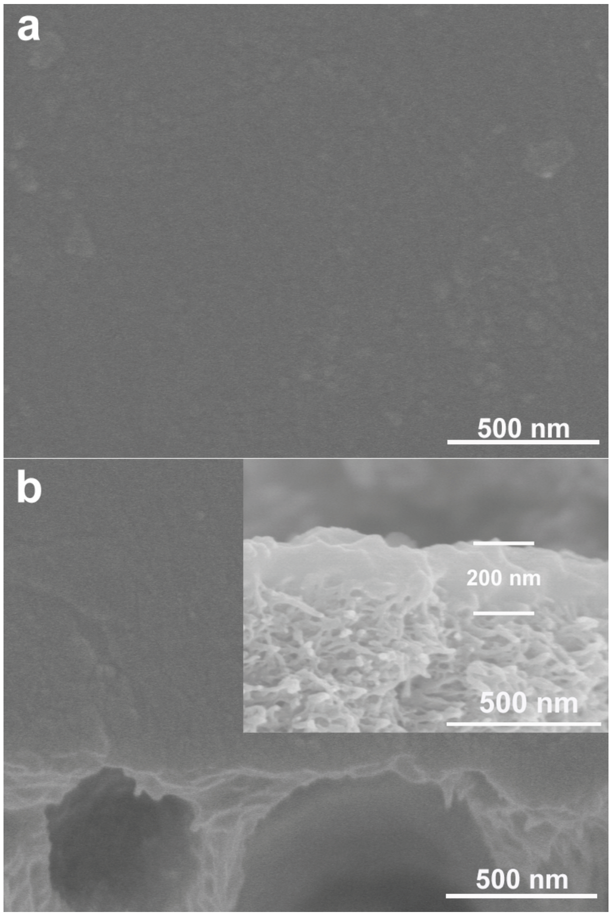

Supplementary Figure 6 │ SEM images. (a) top view and (b) cross section-view SEM images of COF-EB_1BD_3/PAN (inset: the thickness of the membrane).

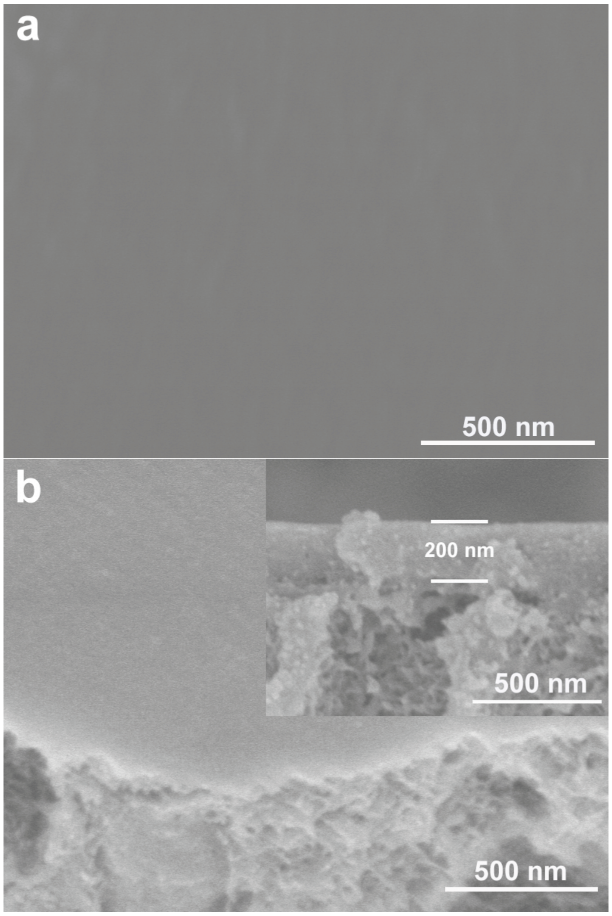

Supplementary Figure 7 │ SEM images. (a) top view and (b) cross section-view SEM images of COF-EB_1BD_2/PAN (inset: the thickness of the membrane).

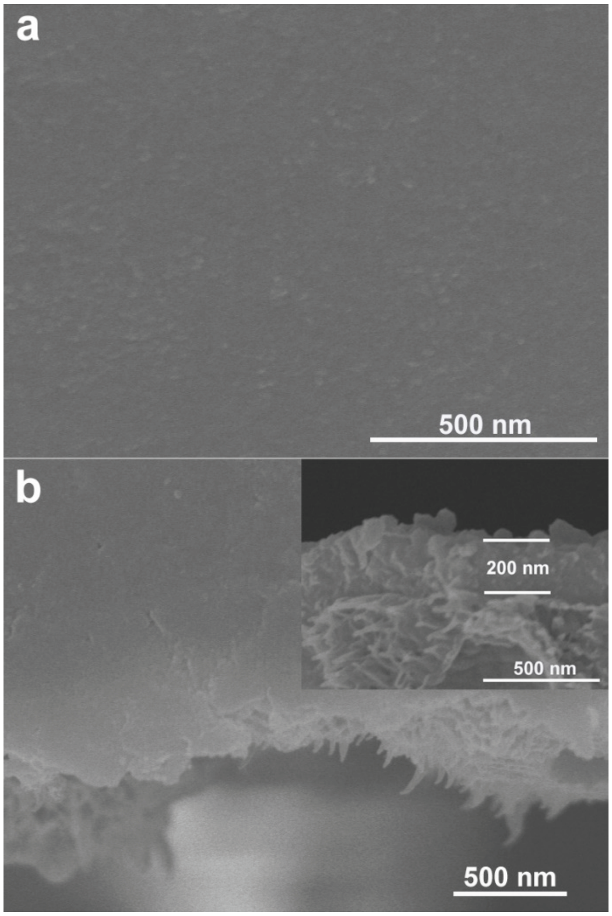

Supplementary Figure 8 | SEM images. (a) top view and (b) cross section-view SEM images of COF-EB/PAN (inset: the thickness of the membrane).

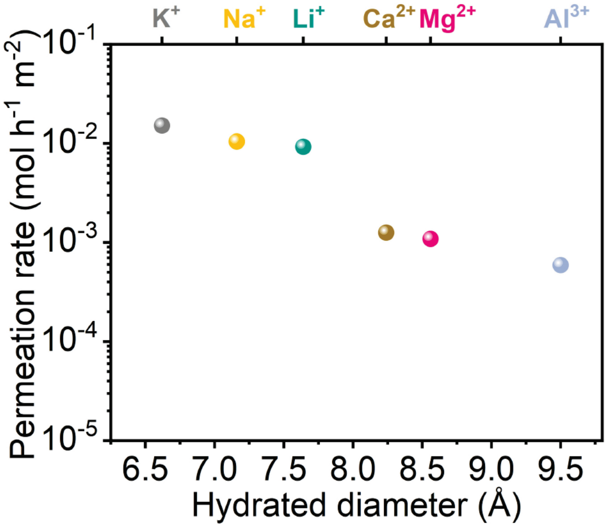

Supplementary Figure 9 | The permeation rates of various salts (0.1 M) through the COF-EB$_1$BD$_2$/PAN membrane. Considering that the pore size of the COF channel is larger than the hydrated ion diameters, the molecular sieving effect can not be used to explain the difference in the ion permeation rates. Given that the impact of ion valence on the permeation rate is greater than their hydrated radius, we rationalized the trend of ion transport to the Donnan membrane equilibrium. Electrostatic forces from the charged sites on membranes cause counter-ions to move in the direction of their concentration gradient. In combination with the requirement of electroneutrality, there is a competition between electrostatic repulsion of co-ions against the charged nanochannels and the electrostatic attraction from counter-ions filled in the nanochannels. Given a balanced electrostatic interaction with monovalent co-ions and counter-ions, the transport rates of the K^+, Na^+, and Li^+ are greater than that of Ca^{2+}, Mg^{2+}, and Al^{3+}. The slight difference in the permeation rate between the ions with the same valence can be reasoned by their different intrinsic mobility.

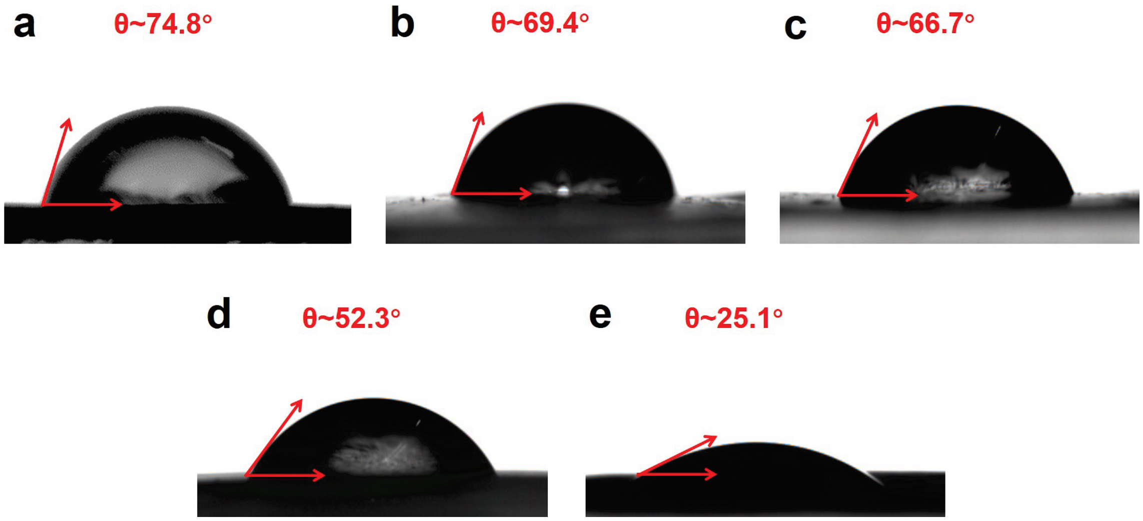

Supplementary Figure 10 | Photographs of water droplets on the COF-EB_xBD_y/PAN membranes. (a) COF-BD/PAN, (b) COF-EB_1BD_5/PAN, (c) COF-EB_1BD_3/PAN, (d) COF-EB_1BD_2/PAN, and (e) COF-EB/PAN.

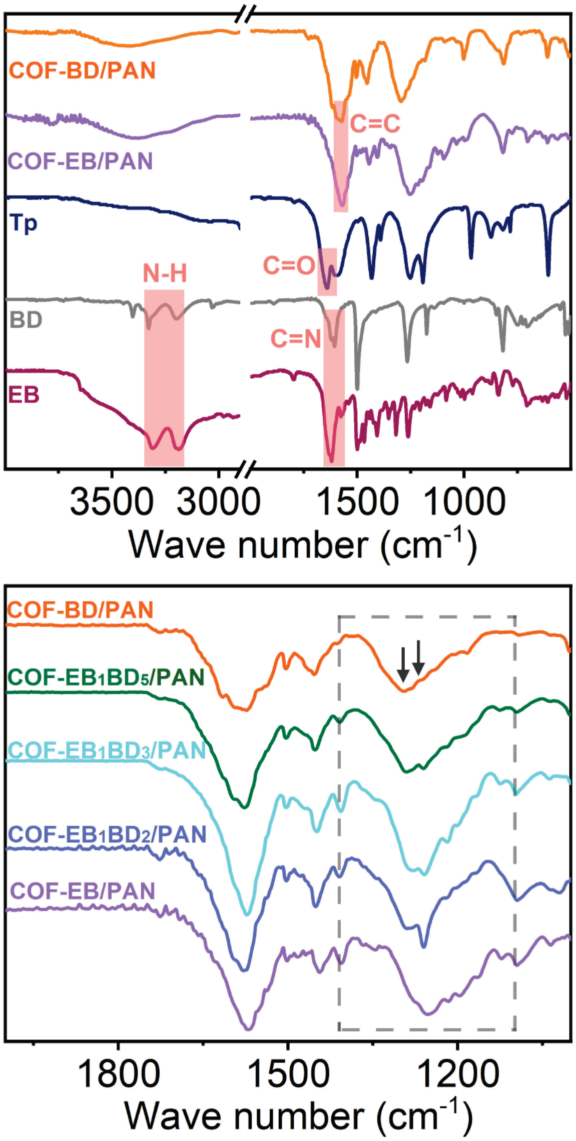

Supplementary Figure 11 | IR spectra.

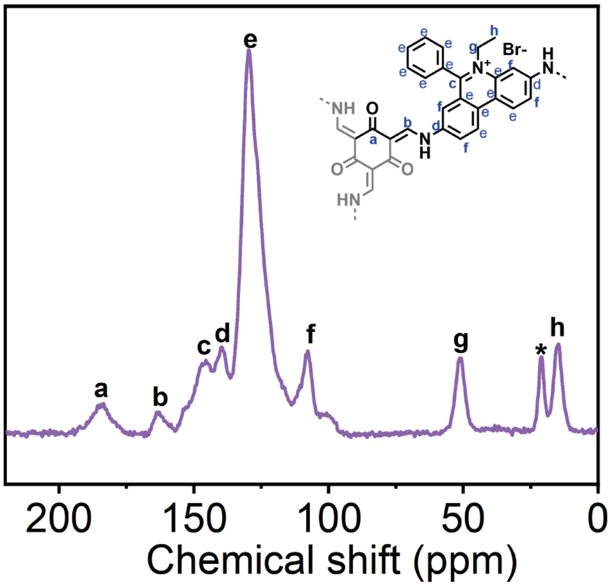

Supplementary Figure 12 | Solid-state ^{13}C NMR spectra for the free-standing COF-EB membrane (* the carbon atom from methanol).

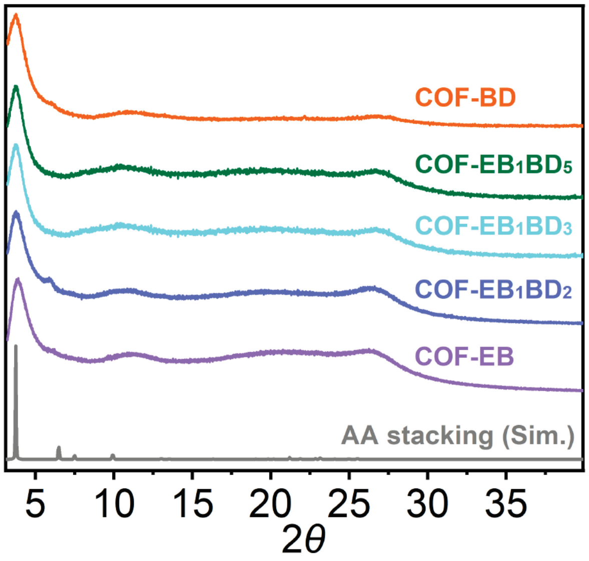

Supplementary Figure 13 | Calculated and experimental PXRD patterns of the free-standing COF-EB$_x$BD$_y$ membranes. The crystallinity of the membranes was confirmed by XRD analysis, which showed a set of peaks that were consistent with the simulated pattern derived from the structure of eclipsed stacked 2D layered sheets with continuous nanometer-scale channels normal to the stacking direction (see the structure in Supplementary Fig. 16).

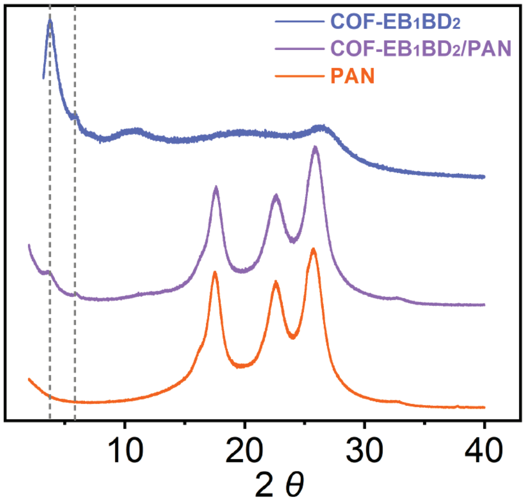

Supplementary Figure 14 | XRD Patterns. Because of the strong background peaks from PAN, peaks from the thin COF layer were largely suppressed. Nonetheless, the position and the relatively intensity of the COF layer on the composite are similar to those of the corresponding free-standing COF membrane, indicative of its crystallinity.

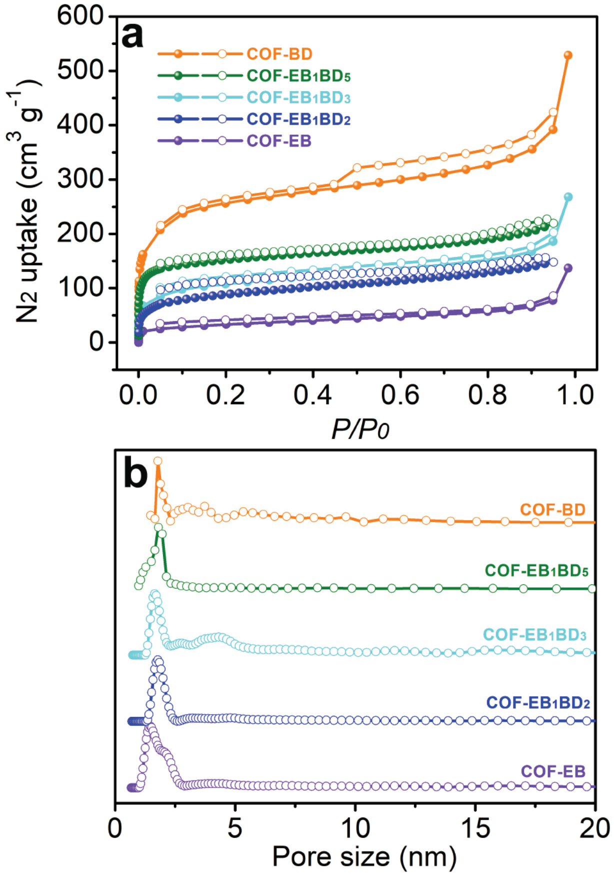

Supplementary Figure 15 │Porosity evaluation. (a) N_2 sorption isotherms collected at 77 K of the free-standing COF-EB_xBD_y membrane and (b) the corresponding pore size distribution based on the NLDFT method. The BET surface area of the free-standing COF-EB_xBD_y membrane was calculated to be 764, 370, 340, 286, and 124 m^2 g^{-1} for COF-BD, COF-EB_1BD_5, COF-EB_1BD_3, COF-EB_1BD_2, and COF-EB, respectively. The pore size distributions for COF-BD, COF-EB_1BD_5, COF-EB_1BD_3, COF-EB_1BD_2, and COF-EB were centered at 1.8, 1.7, 1.6, 1.6, and 1.4 nm, respectively.

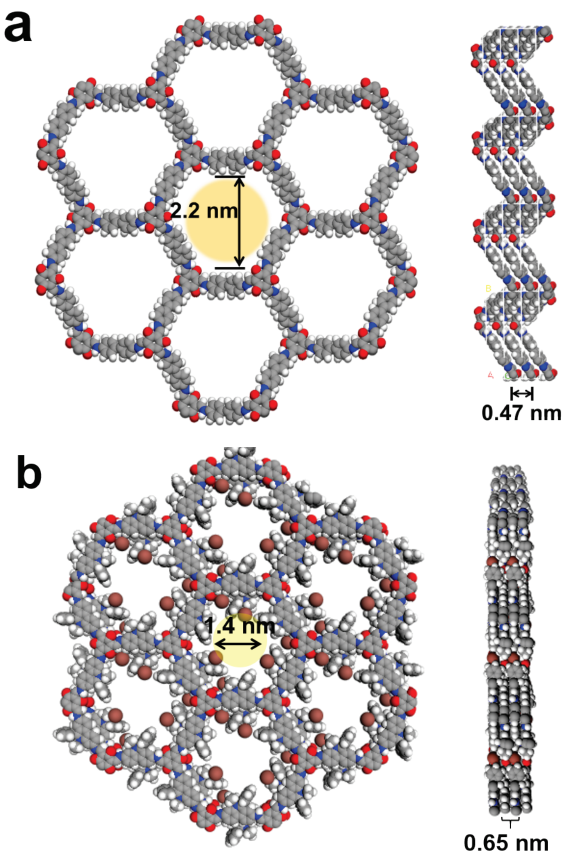

Supplementary Figure 16 | Simulated structures. Graphic view of AA-stacking mode of (a) COF-BD and (b) COF-EB (gray, C; blue, N; red, O; white, H; brown, Br).

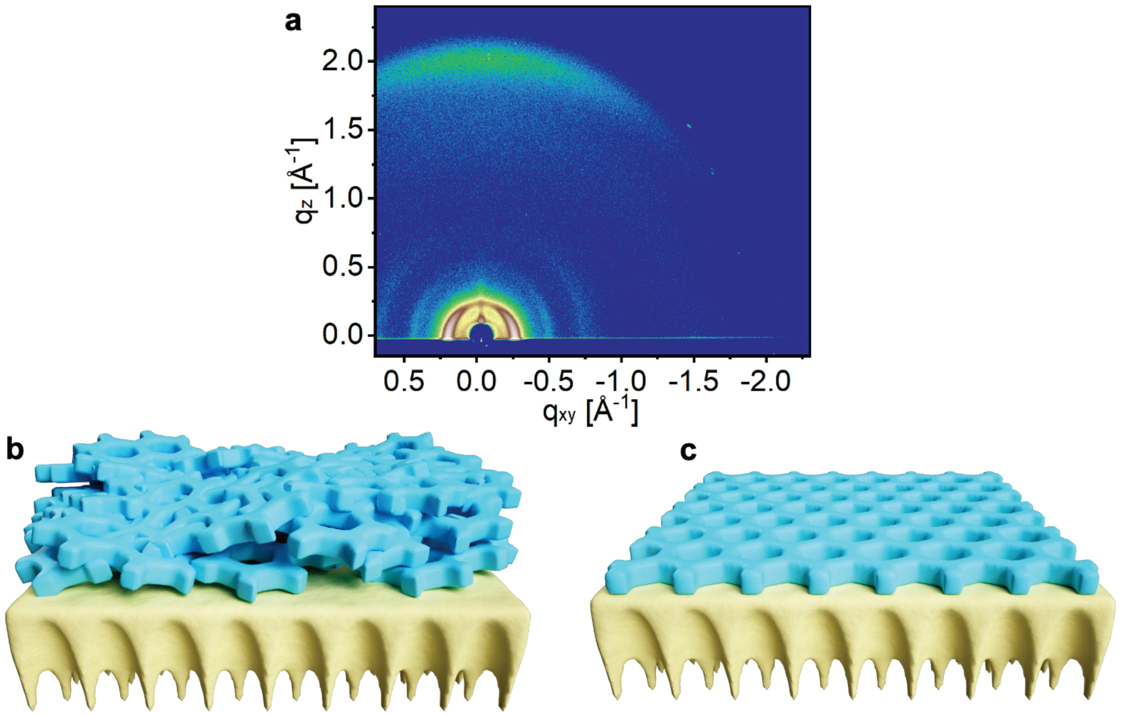

Supplementary Figure 17 | Investigation of the orientation of the COF layer on the PAN support. Grazing-incidence wide-angle X-ray scattering (GIWAXS) patter of COF-EB_1BD_2 (a), which showed that there is a slight preferential orientation of the a-b plane for the COF membrane (parallel to the transport pathway) and schematic illustration of the COF layer on the PAN support with a weak specific preferred orientation (b) and a well specific preferred orientation (c).

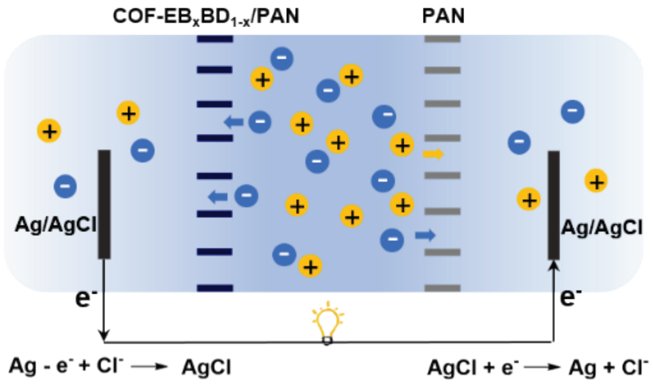

Supplementary Figure 18 | Schematic illustration of the testing setup for the evaluation of permselectivity. A conductive cell segregated by the COF and PAN components into three compartments was used. In this device, the side compartments immersed with Ag/AgCl electrodes were filled with identical low-concentration electrolyte solutions, while the middle compartment was filled with a high-concentration electrolyte solution. Driven by the concentration gradient, anions selectively across the COF-EB$_x$BD$_y$/PAN membrane and accumulate in the low-salinity reservoir, cations remain in the high-salinity reservoir, which is connected with the other low-salinity reservoir and separated by a nonselective PAN membrane to avoid solution quickly mixed while allowing the transport of excess cations. To ensure charge neutrality, redox reactions occur at the electrodes, during which the ionic charge flux is converted into an electrical current.

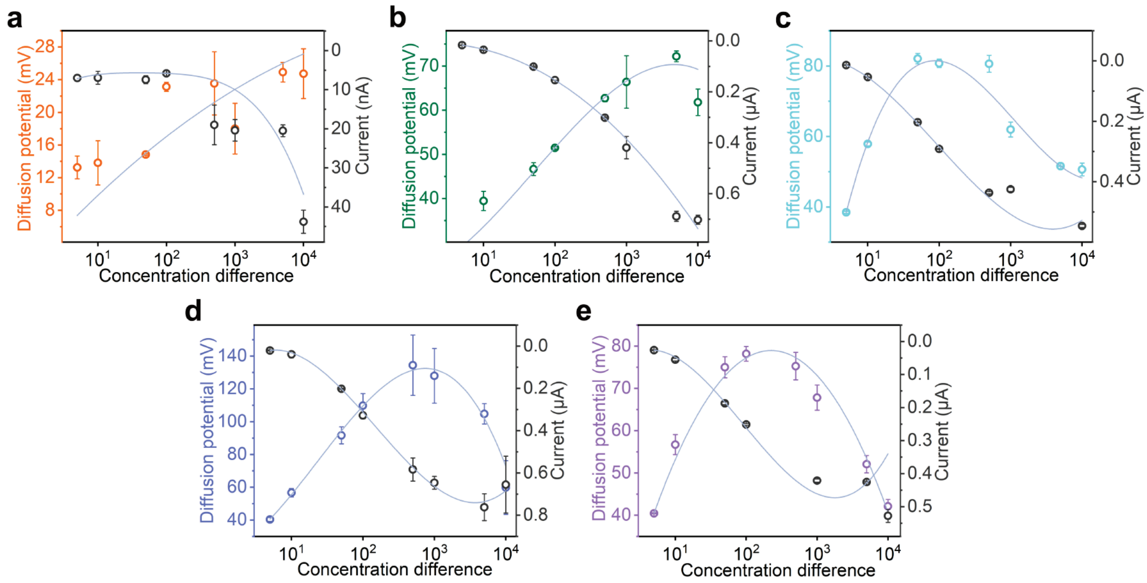

Supplementary Figure 19 | Plots of the recorded V_{oc} and I_{sc} versus KCl concentration difference. (a) COF-BD/PAN, (b) COF-EB_1BD_5/PAN, (c) COF-EB_1BD_3/PAN, (d) COF-EB_1BD_2/PAN, and (e) COF-EB/PAN. Error bars represent standard deviation of three different measurements.

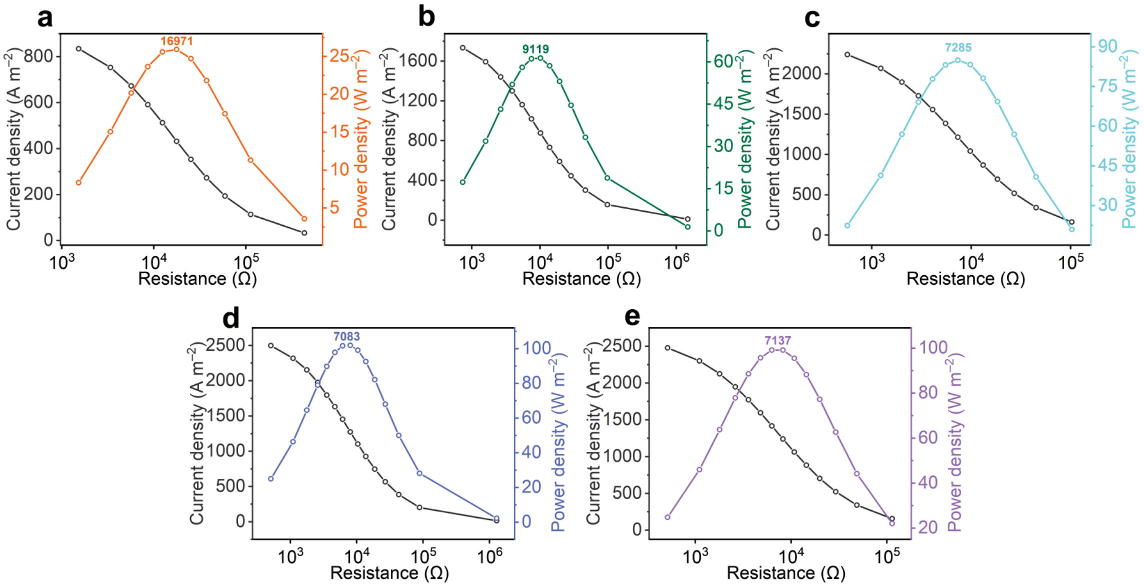

Supplementary Figure 20 │ The diffusion currents and output power densities as a function of load resistances for the COF active layer facing 0.5 M NaCl. (a) COF-BD/PAN, (b) COF-EB_1BD_5/PAN, (c) COF-EB_1BD_3/PAN, (d) COF-EB_1BD_2/PAN, and (e) COF-EB/PAN.

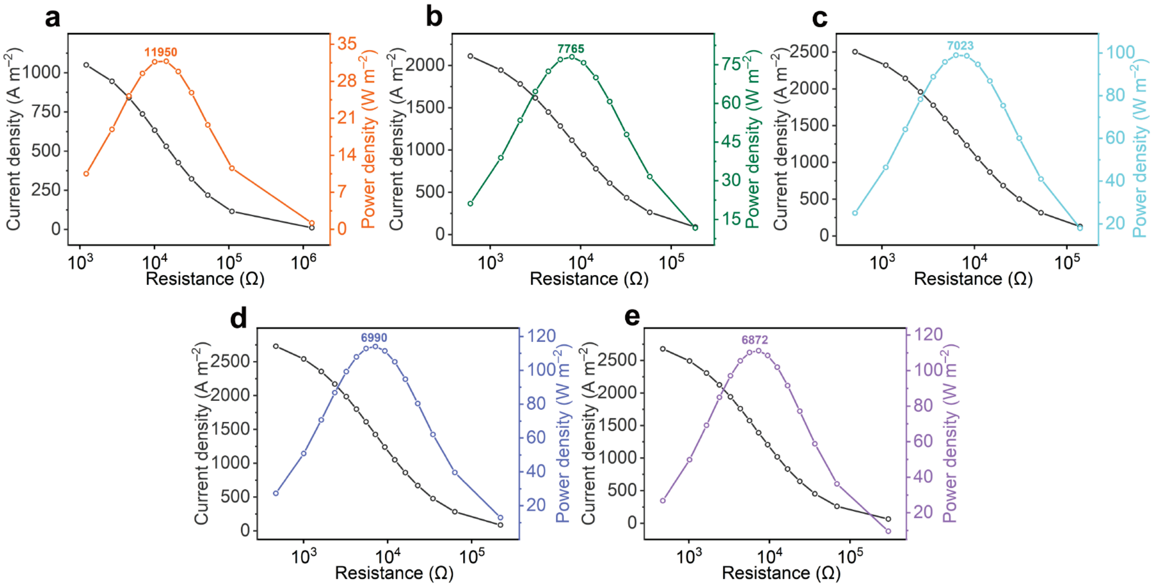

Supplementary Figure 21 | The diffusion currents and output power densities as a function of load resistances for the COF active layer facing 0.01 M NaCl. (a) COF-BD/PAN, (b) COF-EB_1BD_5/PAN, (c) COF-EB_1BD_3/PAN, (d) COF-EB_1BD_2/PAN, and (e) COF-EB/PAN.

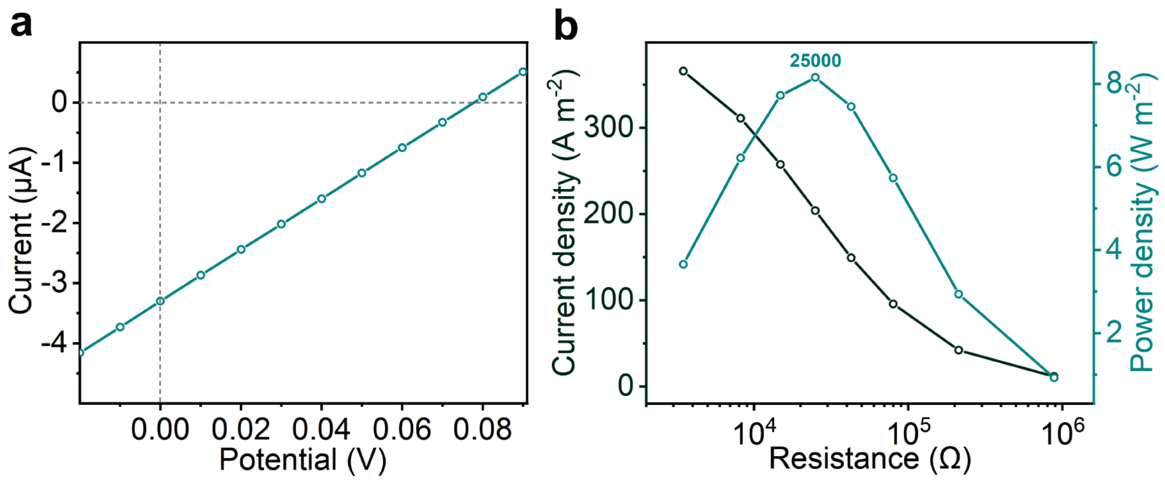

Supplementary Figure 22 | (a) I−V curve and (b) the diffusion current and output power density as a function of load resistance for PAN under a NaCl concentration difference of 0.01/0.5 M. From the intercepts on the current and voltage axes, we can directly obtain the corresponding short-circuit current (I_{sc}) and open-circuit voltage (V_{oc}). The measured V_{oc} actually consists of two parts: the diffusion potential (Φ_{diff}) which is contributed by the selective ion transport and the redox potential (Φ_{redox}) which is generated by the unequal potential drop at the electrode−solution interface. The Φ_{diff} is calculated to be X mV. According to the following equation, where ϕ_{diff} is the diffusion potential, S is effective area, and R_{membr} is the membrane resistance, the P_{diff} is estimated to be 6.2×10^{-4} W m^{-2}.

$$P_{diff} = \frac{{\phi_{diff}}^2}{4SR_{membr}}$$

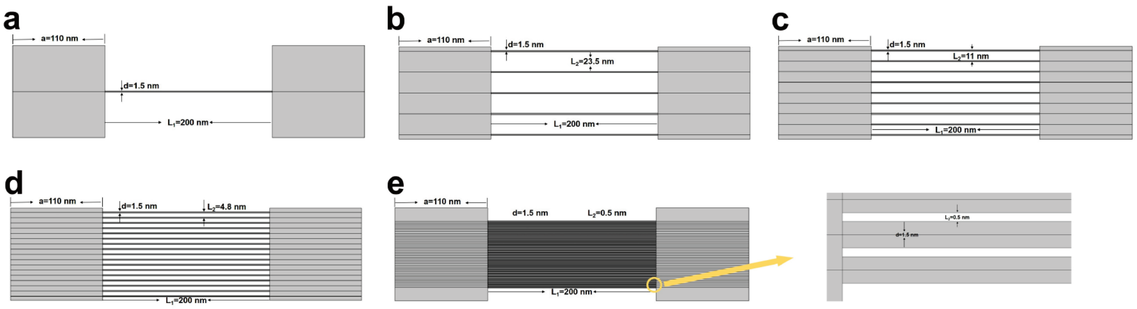

Supplementary Figure 23 | Schematic illustration of 2D model with various channel densities used for calculation under the concentration difference of 0.5 M/0.01 M. (a) 1 channel, (b) 5 channels, (c) 9 channels, (d) 17 channels, and (e) 40 channels.

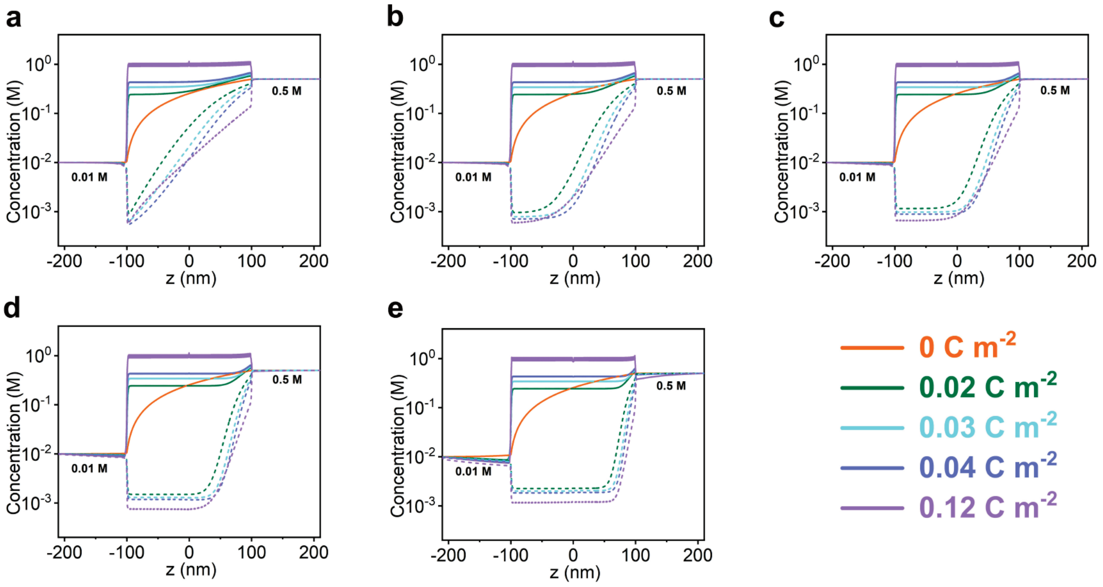

Supplementary Figure 24 │ Numerical simulation of the impact of charge and channel densities on the Na^+ (dashed line) and Cl^- (solid line) distribution in the nanochannels. (a) 1 channel, (b) 5 channels, (c) 9 channels, (d) 17 channels, and (e) 40 channels.

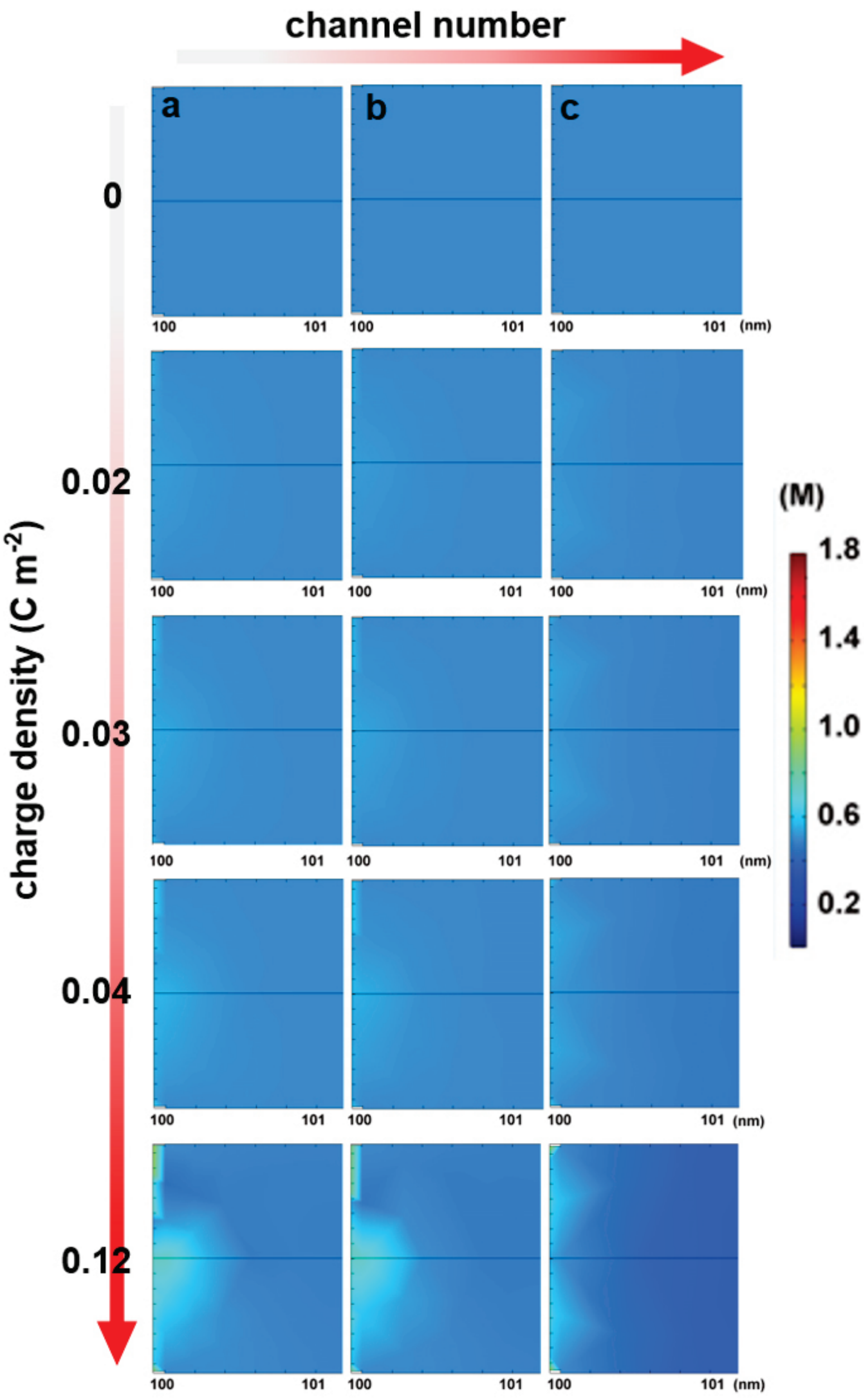

Supplementary Figure 25 | Numerical simulation of the impact of charge and channel densities on the distribution of Cl^- ions at the entrance of pore channels facing 0.5 M NaCl. (a) 1 channel, (b) 9 channels, and (c) 40 channels.

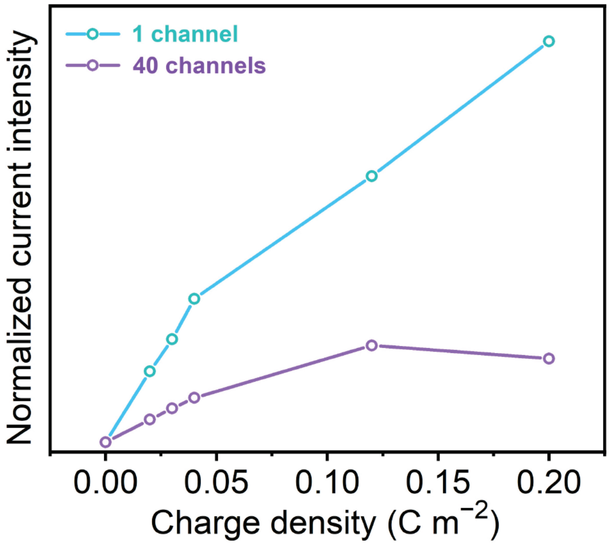

Supplementary Figure 26 │ Numerical simulation of the impact of charge and channel densities on the ion current. Ion flux of multipore membranes obviously deviate from the behavior of single-pore counterparts, unveiling the fact that the high power density estimated from the single nanopores could not be simply achieved with multipore membranes as the pore–pore interactions cannot be ignored at high porosity.

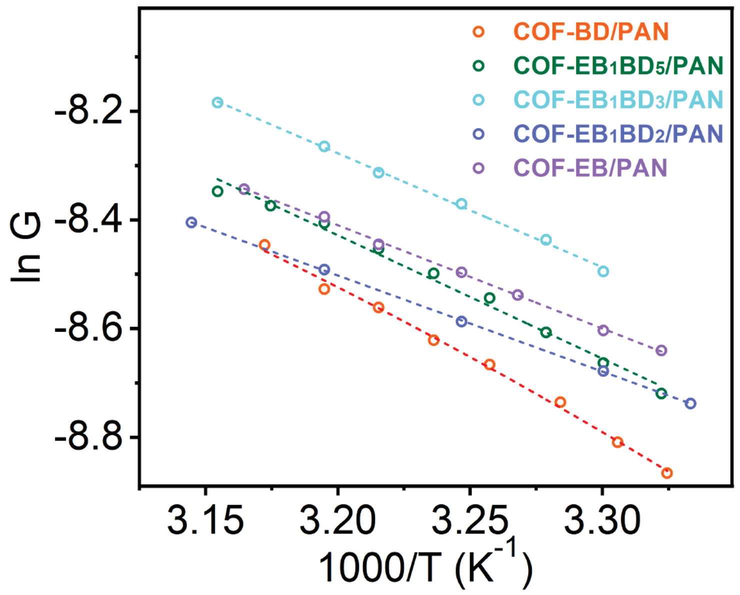

Supplementary Figure 27 | Evaluation of the ion transport energy barrier. Ion conductance curves versus temperature across COF- EB_xBD_y/PAN and the corresponding Arrhenius plots, yielding activation energies of 21.9, 18.9, 17.4, 14.7, and 15.8 kJ mol^{-1} for COF-BD/PAN, COF-EB_1BD_5/PAN, COF-EB_1BD_3/PAN, COF-EB_1BD_2/PAN, and COF-EB/PAN respectively.

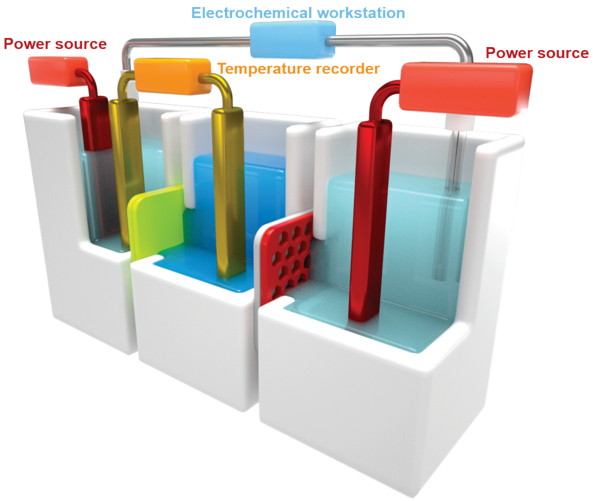

Supplementary Figure 28 | Schematic illustration of the experimental setup used for investigating the thermo-osmotic conversion of COF-EB_xBD_y/PAN.

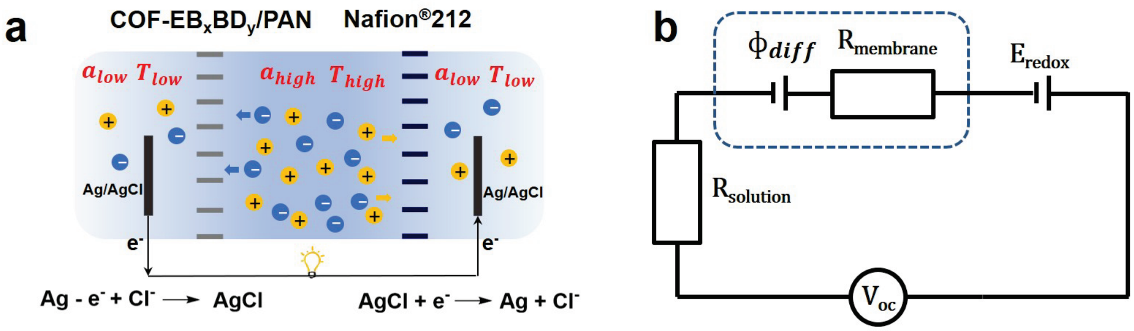

Supplementary Figure 29 | Equivalent circuit. Simplified model of ion transport across COF-EB_xBD_y/PAN and Nafion®212 in the presence of temperature and concentration gradient (left) and equivalent circuit of experimental measurement of thermoelectric response under the open-circuit condition (right).

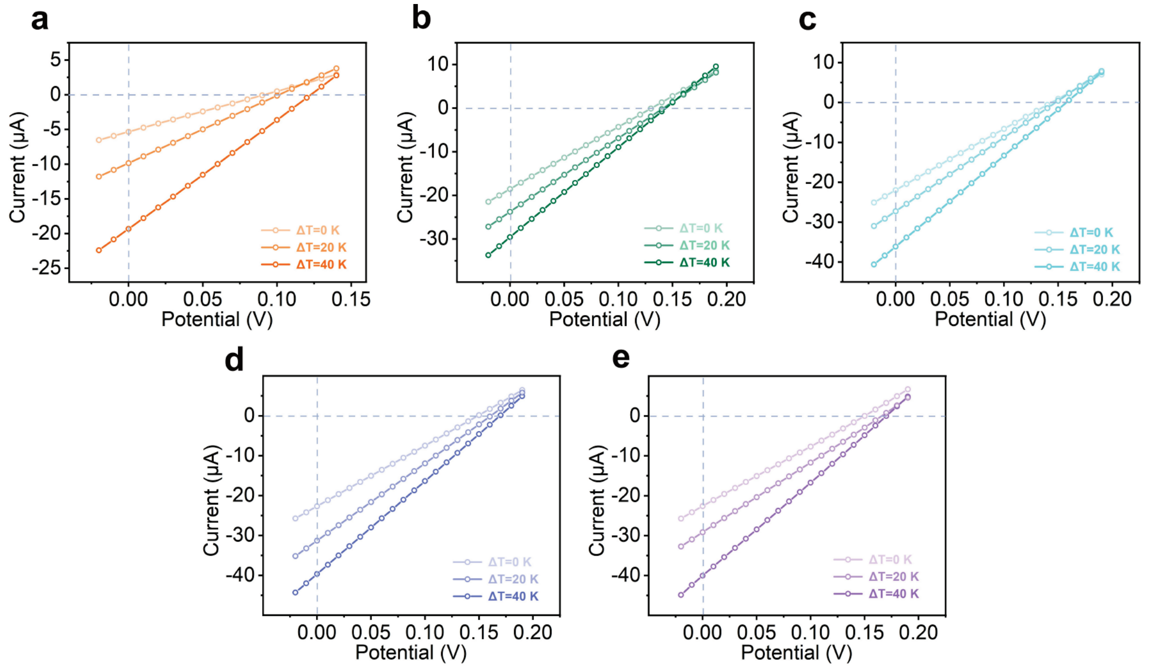

Supplementary Figure 30 | Evaluation of thermo-osmotic conversion performance of COF-EB_xBD_y/PAN. Representative I–V curves of the thermo-osmotic conversion device coupled with COF-EB_xBD_y/PAN and Nafion®212 in the presence of asymmetric NaCl (0.5 M/0.01 M) before and after imposing various temperature gradients for (a) COF-BD/PAN, (b) COF-EB_1BD_5/PAN, (c) COF-EB_1BD_3/PAN, (d) COF-EB_1BD_2/PAN, and (e) COF-EB/PAN, respectively.

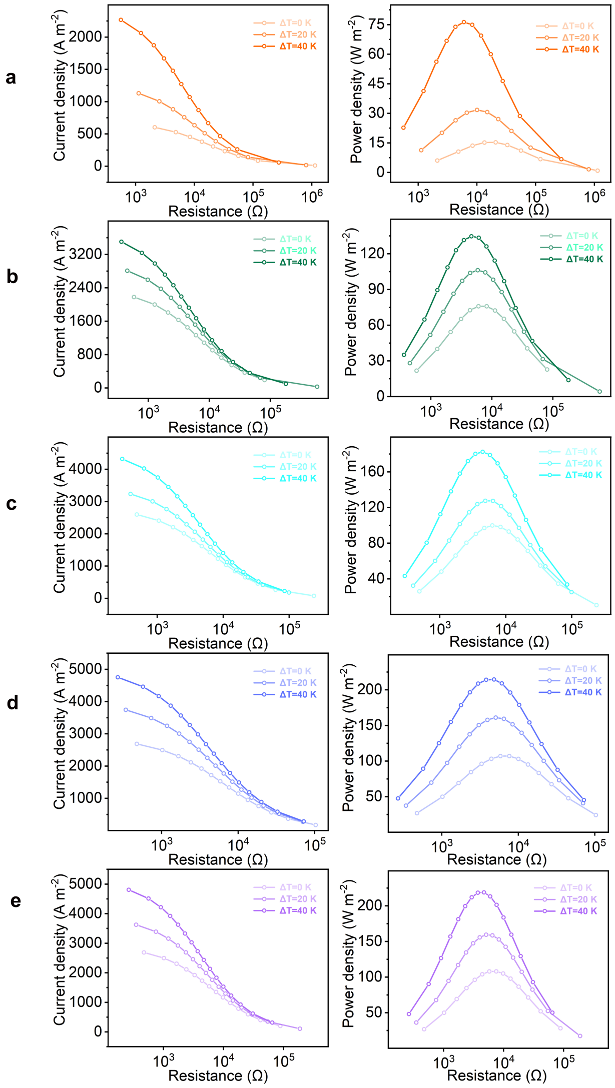

Supplementary Figure 31 | The power was output to an external circuit to supply an electronic load before and after imposing temperature gradients of 20 K and 40 K. (a) COF-BD/PAN, (b) COF-EB_1BD_5/PAN, (c) COF-EB_1BD_3/PAN, (d) COF-EB_1BD_2/PAN, and (e) COF-EB/PAN.

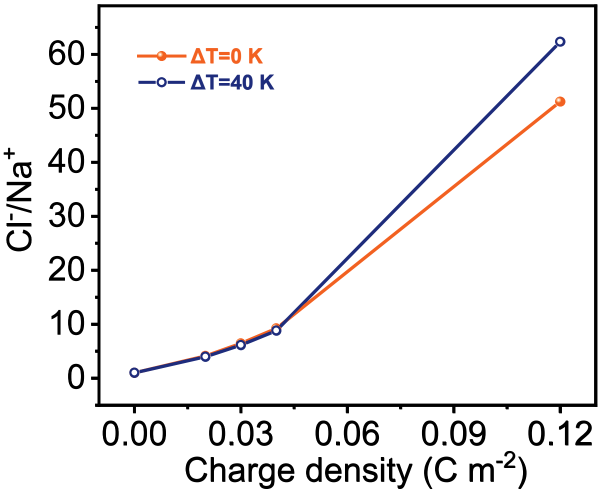

Supplementary Figure 32 | Numerical simulation results. The impact of temperature gradient and charge density on the Cl^-/Na^+ ratio at the mouth of the low-concentration side.

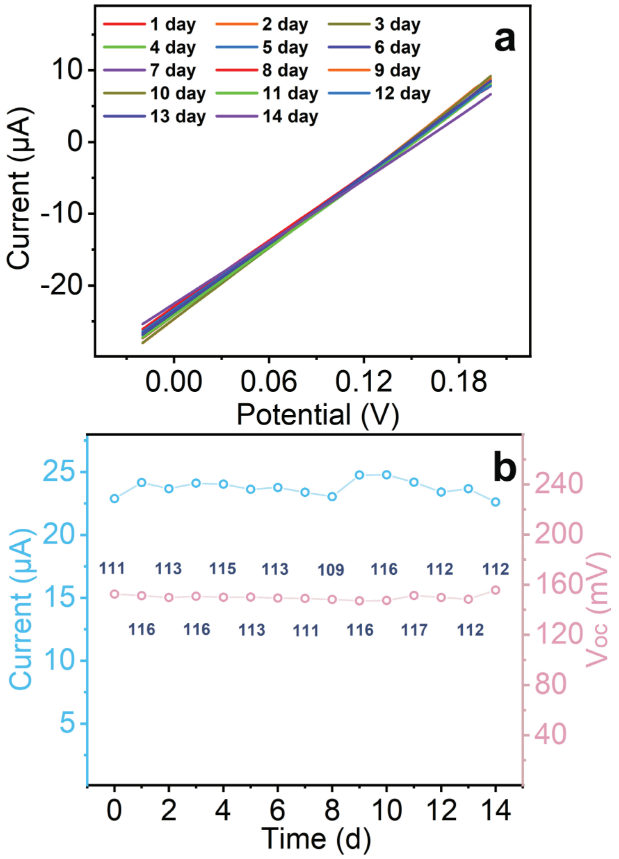

Supplementary Figure 33 | Stability evaluation. (a) Time series of the I−V curves of the thermo-osmotic conversion device constructed by COF-EB_1BD_2/PAN by mixing 0.01 M and 0.5 M NaCl, and (b) plots of V_{oc}, I_{sc}, and output power density of the osmotic conversion device over time.

.

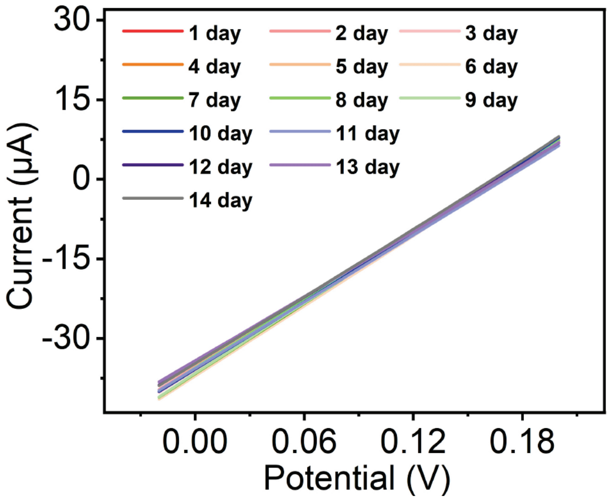

Supplementary Figure 34 | Stability evaluation. Time series of the I−V curves of the thermo-osmotic conversion device constructed by a pair of RED stacks coupled by COF-EB_1BD_2/PAN and Nafion®212 by mixing 0.01 M and 0.5 M NaCl with a temperature difference of 40 K.

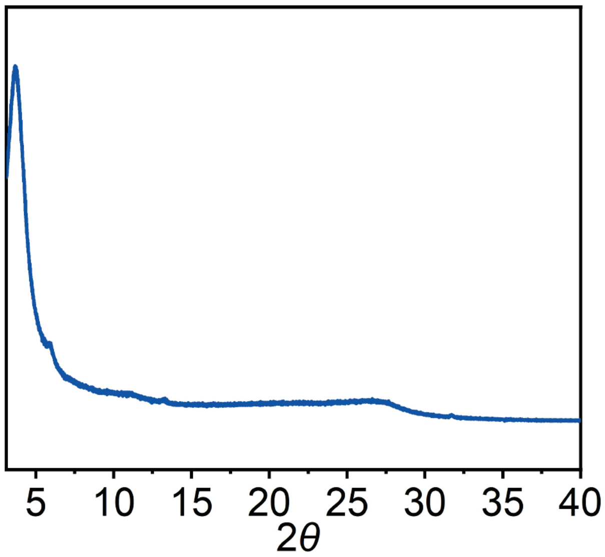

Supplementary Figure 35 | XRD pattern of the free-standing COF-EB$_1$BD$_2$ membrane after being soaked in 0.5 M NaCl for 15 d.

References

1. Li, Z.-Q., Wu, Z.-Q., Ding, X.-L., Wu, M.-Y., & Xia, X.-H. A solar thermoelectric nanofluidic device for solar thermal energy harvesting. *CCS Chem.* **2**, 2174–2182 (2020).
2. Ding, L., Xiao, D., Lu, Z., Deng, J., Wei, Y., Caro, J. & Wang, H. Oppositely charged $Ti_3C_2T_x$ MXene membranes with 2D nanofluidic channels for osmotic energy harvesting. *Angew. Chem. Int. Ed.* **59**, 8720–8726 (2020).
3. Zhang, Z., Yang, S., Zhang, P., Zhang, J., Chen, G. & Feng, X. Mechanically strong MXene/Kevlar nanofiber composite membranes as high-performance nanofluidic osmotic power generators. *Nat. Commun.* **10**, 2920 (2019).
4. Hong, S., Ming, F., Shi, Y., Li, R., Kim, I. S., Tang, C. Y., Alshareef, H. N. & Wang, P. Two-dimensional $Ti_3C_2T_x$ MXene membranes as nanofluidic osmotic power generators. *ACS Nano* **13**, 8917 (2019).
5. Yang, G., Liu, D., Chen, C., Qian, Y., Su, Y., Qin, S., Zhang, L., Wang, X., Sun, L. & Lei, W. Stable $Ti_3C_2T_x$ MXene-boron nitride membranes with low internal resistance for enhanced salinity gradient energy harvesting. *ACS Nano* **15**, 6594–6603 (2021).
6. Ji, J., Kang, Q., Zhou, Y., Feng, Y., Chen, X., Yuan, J., Guo, W., Wei, Y. & Jiang, L. Osmotic power generation with positively and negatively charged 2D nanofluidic membrane pairs. *Adv. Funct. Mater.* **27**, 1603623 (2017).
7. Zhang, Z., Shen, W., Lin, L., Wang, M., Li, N., Zheng, Z., Liu, F. & Cao, L. Vertically transported graphene oxide for high-performance osmotic energy conversion. *Adv. Sci.* **7**, 2000286 (2020).
8. Fu, Y., Guo, X., Wang, Y., Wang, X. & Xue, J. An atomically-thin graphene reverse electrodialysis system for efficient energy harvesting from salinity gradient. *Nano Energy* **57**, 783–790 (2019).
9. Xin, W., Xiao, H., Kong, X.-Y., Chen, J., Yang, L., Niu, B., Qian, Y., Teng, Y., Jiang, L. & Wen, L. Biomimetic nacre-like silk-crosslinked membranes for osmotic energy harvesting. *ACS Nano* **14**, 9701–9710 (2020).
10. Zhu, X., Zhou, Y., Hao, J., Bao, B., Bian, X., Jiang, X., Pang, J., Zhang, H., Jiang, Z. & Jiang, L., A charge-density-tunable three/two-dimensional polymer/graphene oxide heterogeneous nanoporous membrane for ion transport. *ACS Nano* **11**, 10816–10824 (2017).
11. Xiao, K., Giusto, P., Wen, L., Jiang, L. & Antonietti, M. Nanofluidic ion transport and energy conversion through ultrathin free-standing polymeric carbon nitride membranes. *Angew. Chem. Int. Ed.* **57**, 10123–10126 (2018).
12. Chen, C., Liu, D., He, L., Qin, S., Wang, J., Razal, J. M., Kotov, N. A. & Lei, W. Bio-inspired nanocomposite membranes for osmotic energy harvesting. *Joule* **4**, 247–261 (2020).
13. Pendse, A., Cetindag, S., Rehak, P., Behura, S., Gao, H., Nguyen, N. H. L., Wang, T., Berry, V., Král, P., Shan, J. & Kim, S., Highly efficient osmotic energy harvesting in charged boron-nitride-nanopore membranes. *Adv. Funct. Mater*. **31**, 2009586 (2021).
14. Siria, A., Poncharal, P., Biance, A.-L., Fulcrand, R., Blasé, X., Purcell, S. T. & Bocquet, L. Giant osmotic energy conversion measured in a single transmembrane boron nitride nanotube. *Nature* **494**, 455–458 (2013).
15. Xin, W., Zhang, Z., Huang, X., Hu, Y., Zhou, T., Zhu, C., Kong, X.-Y., Jiang, L. & Wen, L. High-performance silk-based hybrid membranes employed for osmotic energy conversion. *Nat. Commun.* **10**, 3876 (2019).
16. Gao, J., Guo, W., Feng, D., Wang, H., Zhao, D. & Jiang, L. High-performance ionic diode membrane for salinity gradient power generation. *J. Am. Chem. Soc.* **136**, 12265–12272 (2014).
17. Sui, X., Zhang, Z., Li, C., Gao, L., Zhao, Y., Yang, L., Wen, L. & Jiang, L. Engineered nanochannel membranes with diode-like behavior for energy conversion over a wide pH range. *ACS Appl. Mater. Interfaces.* **11**, 23815–23821 (2019).
18. Xie, L., Zhou, S., Liu, J., Qiu, B., Liu, T., Liang, Q., Zheng, X., Li, B., Zeng, J., Yan, M., He, Y., Zhang, X., Zeng, H., Ma, D., Chen, P., Liang, K., Jiang, L., Wang, Y., Zhao, D. & Kong, B. Sequential superassembly of nanofiber arrays to carbonaceous ordered mesoporous nanowires and their heterostructure membranes for osmotic energy conversion. *J. Am. Chem. Soc.* **143**, 6922–6932 (2021).
19. Zhou, S., Xie, L., Zhang, L., Wen, L., Tang, J., Zeng, J., Liu, T., Peng, D., Yan, M., Qiu, B., Liang, Q., Liang, K., Jiang, L. & Kong, B. Interfacial super-assembly of ordered mesoporous silica-alumina heterostructure membranes with pH-sensitive properties for osmotic energy harvesting. *ACS Appl. Mater. Interfaces* **13**, 8782–8793 (2021).
20. Van Toan, N., Hasnan, M. M. I. M., Udagawa, D., Inomata, N., Toda, M., Said, S. M., Sabri, M. F. M. & Ono, T. Thermoelectric power battery using al_2o_3 nanochannels of 10 nm diameter for energy harvesting of low-grade waste heat. *Energy Convers. Manag.* **199**, 111979 (2019).
21. Li, Z.-Q., Wu, Z.-Q., Ding, X.-L., Wu, M.-Y. & Xia, X.-H. A solar thermoelectric nanofluidic device for solar thermal energy harvesting. *CCS Chem*. **2**, 2174–2182 (2020).
22. Chen, K., Yao, L., Yan, F., Liu, S., Yang, R. & Su, B. Thermo-osmotic energy conversion and storage by nanochannels. *J. Mater. Chem. A* **7**, 25258–25261 (2019).
23. Kim, D.-K., Duan, C., Chen, Y.-F. & Majumdar, A. Power generation from concentration gradient by reverse electrodialysis in ion-selective nanochannels. *Microfluid. Nanofluid.* **9**, 1215–1224 (2010).

24. Yan, F., Yao, L., Chen, K., Yang, Q. & Su, B. An untrathin and highly porous silica nanochannel membrane: toward highly efficient salinity energy conversion. *J. Mater. Chem. A.* **7**, 2385–2391 (2019).
25. Ouyang, W., Wang, W., Zhang, H., Wu, W. & Li, Z. Nanofluidic crystal: a facile, high-efficiency and high-power-density scaling up scheme for energy harvesting based on nanofluidic reverse electrodialysis. *Nanotechnology* **24**, 345401 (2013).
26. Hwang, J., Kataoka, S., Endo, A. & Daiguji, H. Enhanced energy harvesting by concentration gradient-driven ion transport in SBA-15 mesoporous silica thin films. *Lab Chip*. **16**, 3824–3832 (2016).
27. Zhang, Z., Zhang, P., Yang, S., Zhang, T., Löffler, M., Shi, H., Lohe, M. R. & Feng, X. Oxidation promoted osmotic energy conversion in black phosphorus membranes. *Proc. Natl Acad. Sci. USA* **117**, 13959–13966 (2020).
28. Zhu, C., Liu, P., Niu, B., Liu, Y., Xin, W., Chen, W., Kong, X.-Y., Zhang, Z., Jiang, L. & Wen, L. Metallic two-dimensional MoS_2 composites as high-performance osmotic energy conversion membranes. *J. Am. Chem. Soc.* **143**, 1932–1940 (2021).
29. Feng, J., Graf, M., Liu, K., Ovchinnikov, D., Dumcenco, D., Heiranian, M., Nandigana, V., Aluru, N. R., Kis, A. & Radenovic, A. Single-layer MoS_2 nanopores as nanopower generators. *Nature* **536**, 197–200 (2016).
30. Wu, C., Xiao, T., Tang, J., Zhang, Q., Liu, Z., Liu, J. & Wang, H. Biomimetic temperature-gated 2D cationic nanochannels for controllable osmotic power harvesting. *Nano Energy* **76**, 105113 (2020).
31. Cheng, H., Zhou, Y., Feng, Y., Geng, W., Liu, Q., Guo, W. & Jiang, L. Electrokinetic energy conversion in self-assembled 2D nanofluidic channels with janus nanobuilding blocks. *Adv. Mater.* **29**, 1700177 (2017).
32. Zhu, X., Hao, J., Bao, B., Zhou, Y., Zhang, H., Pang, J., Jiang, Z. & Jiang, L. Unique ion rectification in hypersaline environment: a high-performance and sustainable power generator system. *Sci. Adv.* **4**, eaau1665 (2018).
33. Yeh, L.-H., Huang, Z.-Y., Liu, Y.-C., Deng, M.-J., Chou, T.-H., OuYang, H.-C., Ahamad, T., Alshehrif, S. M. & Wu, K. C.-W. A nanofluidic osmotic power generator demonstrated in polymer gel electrolytes with substantially enhanced performance. *J. Mater. Chem. A.* **7**, 26791–26796 (2019).
34. Zhang, Z., He, L., Zhu, C., Qian, Y., Wen, L. & Jiang, L. Improved osmotic energy conversion in heterogeneous membrane boosted by three-dimensional hydrogel interface. *Nat. Commun.* **11**, 875 (2020).
35. Xu, Y., Song, Y. & Xu, F. TEMPO oxidized cellulose nanofibers-based heterogenous membrane employed for concentration-gradient-driven energy harvesting. *Nano Energy* **79**, 105468 (2021).
36. Zhang, Z., Sui, X., Li, P., Xie, G., Kong, X.-Y., Xiao, K., Gao, L., Wen, L. & Jiang, L. Ultrathin and ion-selective Janus membranes for high-performance osmotic energy conversion. *J. Am. Chem. Soc.* **139**, 8905–8914 (2017).
37. Guo, B. W., Cao, L., Xia, J., Nie, F.-Q., Ma, Wen., Xue, J., Song, Y., Zhu, D., Wang, Y. & Jiang, L. Energy harvesting with single-ion-selective nanopores: a concentration-gradient-driven nanofluidic power source. *Adv. Funct. Mater.* **20**, 1339–1344 (2010).
38. Chang, H.-K., Choi, E. & Park, J. Paper-based energy harvesting from salinity gradients. *Lab Chip.* **16**, 700–708 (2016).
39. Zhang, Z., Kong, X.-Y., Xiao, K., Liu, Q., Xie, G., Li, P., Ma, J., Tian, Y., Wen, L. & Jiang, L. Engineered asymmetric heterogeneous membrane: a concentration-gradient-driven energy harvesting device. *J. Am. Chem. Soc.* **137**, 144765–14772 (2015).
40. Chen, W., Zhang, Q., Qian, Y., Xin, W., Hao, D., Zhao, X., Zhu, C., Kong, X.-Y., Lu, B., Jiang, L. & Wen, L. Improved ion transport in hydrogel-based nanofluidics for osmotic energy conversion. *ACS Cent. Sci.* **6**, 2097–2104 (2020).
41. Yu, C., Zhu, X., Wang, C., Zhou, Y., Jia, X., Jiang, L., Liu, X. & Wallace, G. G. A smart cyto-compatible asymmetric polypyrrole membrane for salinity power generation. *Nano Energy* **53**, 475–482 (2018).
42. Sun, Y., Dong, T., Lu, C., Xin, W., Yang, L., Liu, P., Qian, Y., Zhao, Y., Kong, X.-Y., Wen, L. & Jiang, L. Tailoring a poly (ether sulfone) bipolar membrane: osmotic-energy generator with high power density. *Angew. Chem. Int. Ed.* **59**, 17423–17428 (2020).
43. Huang, X., Zhang, Z., Kong, X.-Y., Sun, Y., Zhu, C., Liu, P., Pang, J., Jiang, L. & Wen, L. Engineered PES/SPES nanochannel membrane for salinity gradient power generation. *Nano Energy* **59**, 354–362 (2019).
44. Lin, C.-Y., Combs, C., Su, Y.-S., Yeh, L.-H. & Siwy, Z. S. Rectification of concentration polarization in mesopores leads to high conductance ionic diodes and high performance osmotic power. *J. Am. Chem. Soc.* **141**, 3691–3698 (2019).
45. Zhao, Y., Wang, J., Kong, X.-Y., Xin, W., Zhou, T., Qian, Y., Yang, L., Pang, J., Jiang, L. & Wen, L. Robust sulfonated poly (ether ether ketone) nanochannels for high-performance osmotic energy conversion. *Natl. Sci. Rev.* **7**, 1349–1359 (2020).
46. Li, C., Wen, L., Sui, X., Cheng, Y., Gao, L. & Jiang, L. Large-scale, robust mushroom-shaped nanochannel array membrane for ultrahigh osmotic energy conversion. *Sci. Adv*. **7**, eabg2183 (2021).
47. Mai, V.-P. & Yang, R.-J. Boosting power generation from salinity gradient on high-density nanoporous membrane using thermal effect. *Applied Energy* **274**, 115294 (2020).
48. Long, R., Luo, Z., Kuang, Z., Liu, Z. & Liu, W. Effects of heat transfer and the membrane thermal conductivity on the thermally nanofluidic salinity gradient energy conversion. *Nano Energy* **67**, 104284 (2020).
49. Sun, Y., Wu, Y., Hu, Y., Zhu, C., Guo, H., Kong, X.-Y., Luo, E., Jiang, L. & Wen, L. Thermoenhanced osmotic power generator

via lithium bromide and asymmetric sulfonated poly(ether ether ketone)/poly(ether sulfone) nanofluidic membrane. *NPG Asia Mater.* **13**, 50 (2021).
50. Turek, M. & Bandura, B. Renewable energy by reverse electrodialysis. *Desalination* **205**, 67–74 (2007).
51. Suda, F., Matsuo, T. & Ushioda, D. Transient changes in the power output from the concentration different cell (dialytic battery) between seawater and river water. *Energy* **32**, 165–173 (2007).
52. Veerman, J., de Jong, R. M., Saakes, M., Metz, S. J. & Harmsen, G. J. Reverse electrodialysis: comparison of six commercial membrane pairs on the thermodynamic efficiency and power density. *J. Membr. Sci.* **343**, 7–15 (2009).
53. Yip, N. Y., Vermaas, D. A., Nijmeijer, K. & Elimelech, M. Thermodynamic, energy efficiency, and power density analysis of reverse electrodialysis power generation with natural salinity gradients. *Environ. Sci. Technol.* **48**, 4925–4936 (2014).
54. Palenzuela, P., Micari, M., Ortega-Delgado, B., Giacalone, F., Zaragoza, G., Alarcón-Padilla, D., Cipollina, A., Tamburini, A. & Micale, G., Performance analysis of a red-med salinity gradient heat engine. *Energies* **11**, 3385 (2018).
55. Li, R., Jiang, J., Liu, Q., Xie, Z. & Zhai, J. Hybrid nanochannel membrane based on polymer/MOF for high-performance salinity gradient power generation. *Nano Energy* **53**, 643–649 (2018).
56. Hou, S., Ji, W., Chen, J., Teng, Y., Wen, L. & Jiang, L. Free-standing covalent organic framework membrane for high-efficiency salinity gradient energy conversion. *Angew. Chem. Int. Ed.* **60**, 9925–9930 (2021).
57. Liu, Y. C., Yeh, L. H., Zheng, M. J. & Wu, K. C. W. Highly selective and high-performance osmotic power generators in subnanochannel membranes enabled by metal-organic frameworks. *Sci. Adv.* **7**, eabe9924 (2021).
58. Man, Z., Safaei, J., Zhang, Z., Wang, Y., Zhou, D., Li, P., Zhang, X., Jiang, L. & Wang, G. Serosa-mimetic nanoarchitecture membranes for highly efficient osmotic energy generation. *J. Am. Chem. Soc.* **143**, 16206–16216 (2021).
59. Chen, J., Xin, W., Kong, X.-Y., Qian, Y., Zhao, X., Chen, W., Sun, Y., Wu, Y., Jiang, L. & Wen, L. Ultrathin and robust silk fibroin membrane for high-performance osmotic energy conversion. *ACS Energy Lett.* **5**, 742–748 (2020).
60. Wu, Z., Ji, P., Wang, B., Sheng, N., Zhang, M., Chen, S. & Wang, H. Oppositely charged aligned bacterial cellulose biofilm with nanofluidic channels for osmotic energy harvesting. *Nano Energy* **80**, 105554 (2021).
61. Wu, Q.-Y., Wang, C., Wang, R., Chen, C., Gao, J., Dai, J., Liu, D., Lin, Z. & Hu, L. Salinity-gradient power generation with ionized wood membranes. *Adv. Energy Mater*. **10**, 1902590 (2020).
62. Zhang, T., Li, K., Zhang, J., Chen, M., Wang, Z., Ma, S., Zhang, N. & Wei, L. High-performance, flexible, and ultralong. crystalline thermoelectric fibers. *Nano Energy* **41**, 35–42 (2017).
63. Zhao, M., Kim, D., Nguyen, V. L., Jiang, J., Sun, L., Lee, Y. H. & Yang, H. Coherent thermoelectric power from graphene quantumdots. *Nano Lett.* **19**, 61–68 (2019).
64. Sun, T., Zhou, B., Zheng, Q., Wang, L., Jiang, W. & Snyder, G. J. Stretchable fabric generates electric power from woven thermoelectric fibers. *Nat. Commun*. **11**, 572 (2020).
65. Cheng, H. & Ouyang, J. Ultrahigh thermoelectric power generation from both ion diffusion by temperature fluctuation and hole accumulation by temperature gradient. *Adv. Energy Mater.* **10**, 2001633 (2020).
66. Peng, J., Witting, I., Geisendorfer, N., Wang, M., Chang, M., Jakus, A., Kenel, C., Yan, X., Shah, R., Snyder, G. J. & Grayson, M. 3D extruded composite thermoelectric threads for flexible energy harvesting. *Nat. Commun.* **10**, 5590 (2019).
67. Qin, B., Wang, D., Liu, X., Qin, Y., Dong, J.-F., Luo, J., Li, J.-W., Liu, W., Tan, G., Tang, X., Li, J.-F., He, J. & Zhao, L.-D. Power generation and thermoelectric cooling enabled by momentum and energy multiband alignments. *Science* **373**, 556–561 (2021).
68. He, W., Wang, D., Wu, H., Xiao, Y., Zhang, Y., Liu, C., Ohta, M., Pennycook, S. J., He, J., Li, J.-F. & Zhao, L.-D. High thermoelectric performance in low-cost $SnS_{0.91}Se_{0.09}$ crystals. *Science* **365**, 1418–1424 (2019).
69. Bu, Z., Zhang, X., Hu, Y., Chen, Z., Lin, S., Li, W., Xiao, C. & Pei, Y. A record thermoelectric efficiency in tellurium-free modules for low-grade waste heat recovery. *Nat Commun.* **13**, 237 (2022).
70. Chen, K., Yao, L. & Su, B. Bionic thermoelectric response with nanochannels. *J. Am. Chem. Soc.* **141**, 8608-8615 (2019).
71. Park, J., Hinckley, A. C., Huang, Z., Chen, G., Yakovenko, A. A., Zou, X. & Bao, Z. High temperature in a Zn-based 3D semiconductive metal-organic framework. *J. Am. Chem. Soc.* **142**, 20531–20535 (2020).
72. Li, Z.-Q., Wu, Z.-Q., Ding, X.-L., Wu, M.-Y. & Xia, X.-H. A solar thermoelectric nanofluidic device for solar thermal energy harvesting. *CCS Chem*. **2**, 2174–2182 (2020).
73. Brown, B. R. Sensing temperature without ion channels. *Nature* **421**, 495 (2003).
74. Zhang, P., Chen, S., Zhu, C., Hou, L., Xian, W., Zuo, X., Zhang, Q., Zhang, L., Ma, S. & Sun, Q. Covalent organic framework nanofluidic membrane as a platform for highly sensitive bionic thermosensation. *Nat. Commun.* **12**, 1844 (2021).
75. Mai, V.-P. & Yang, R.-J. Boosting power generation from salinity gradient on high-density nanoporous membrane using thermal effect. *Appl. Energy* **274**, 115294 (2020).
